# Glucocorticoids Preferentially Influence Expression of Nucleoskeletal Actin Network and Cell Adhesive Proteins in Human Trabecular Meshwork Cells

**DOI:** 10.3389/fcell.2022.886754

**Published:** 2022-04-26

**Authors:** William Bachman, Rupalatha Maddala, Ayon Chakraborty, Camelia Eldawy, Nikolai P. Skiba, Ponugoti V. Rao

**Affiliations:** ^1^ Department of Ophthalmology, Duke University School of Medicine, Durham, NC, United States; ^2^ Department of Pharmacology and Cancer Biology, Duke University School of Medicine, Durham, NC, United States

**Keywords:** glaucoma, intraocular pressure, glucocorticoids, trabecular meshwork, nucleoskeleton, proteomics, transcription, chromatin

## Abstract

Clinical use of glucocorticoids is associated with increased intraocular pressure (IOP), a major risk factor for glaucoma. Glucocorticoids have been reported to induce changes in actin cytoskeletal organization, cell adhesion, extracellular matrix, fibrogenic activity, and mechanical properties of trabecular meshwork (TM) tissue, which plays a crucial role in aqueous humor dynamics and IOP homeostasis. However, we have a limited understanding of the molecular underpinnings regulating these myriad processes in TM cells. To understand how proteins, including cytoskeletal and cell adhesion proteins that are recognized to shuttle between the cytosolic and nuclear regions, influence gene expression and other cellular activities, we used proteomic analysis to characterize the nuclear protein fraction of dexamethasone (Dex) treated human TM cells. Treatment of human TM cells with Dex for 1, 5, or 7 days led to consistent increases (by ≥ two-fold) in the levels of various actin cytoskeletal regulatory, cell adhesive, and vesicle trafficking proteins. Increases (≥two-fold) were also observed in levels of Wnt signaling regulator (glypican-4), actin-binding chromatin modulator (BRG1) and nuclear actin filament depolymerizing protein (MICAL2; microtubule-associated monooxygenase, calponin and LIM domain containing), together with a decrease in tissue plasminogen activator. These changes were independently further confirmed by immunoblotting analysis. Interestingly, deficiency of BRG1 expression blunted the Dex-induced increases in the levels of some of these proteins in TM cells. In summary, these findings indicate that the widely recognized changes in actin cytoskeletal and cell adhesive attributes of TM cells by glucocorticoids involve actin regulated BRG1 chromatin remodeling, nuclear MICAL2, and glypican-4 regulated Wnt signaling upstream of the serum response factor/myocardin controlled transcriptional activity.

## Introduction

The trabecular meshwork, a tissue of mesenchymal origin, plays a crucial role in the modulation of aqueous humor (AH) outflow through the trabecular/conventional pathway, which consists of the trabecular meshwork (TM), juxtacanalicular tissue, and Schlemm’s canal (Tamm, 2009). The volume of AH in the anterior chamber of the eye results from the balance between AH secretion by the ciliary epithelium and outflow/drainage of AH through the trabecular pathway and determines intraocular pressure (IOP) (Stamer and Acott, 2012). However, it is well recognized that relatively the trabecular pathway regulated barrier and filtration activities are crucial for the maintenance of physiological IOP and that impairment in these characteristics of the trabecular pathway increases IOP (Stamer and Acott, 2012). Elevated IOP over the long term is known to induce and enhance optic nerve atrophy and eventually lead to degeneration of the retinal ganglion cells and vision loss in glaucoma patients (Quigley, 2011; Weinreb et al., 2014). Increased IOP is recognized as a predominant risk factor for glaucoma and in particular, lowering of IOP has been found to delay vision loss in glaucoma patients and is a mainstay of glaucoma treatment (Kass et al., 2002; Weinreb et al., 2014). Therefore, there is a great deal of interest in understanding the various molecular pathways regulating AH outflow through the TM to facilitate the development of efficacious and targeted therapies for IOP control in glaucoma patients.

Actin cytoskeletal dynamics, cell adhesive interactions (both cell-extracellular matrix and cell–cell), cellular contractility, extracellular matrix (ECM) accumulation, and mechanical attributes of the trabecular meshwork are recognized to influence AH outflow through the conventional outflow pathway (Raghunathan et al., 2015; Vranka et al., 2015; Filla et al., 2017; Rao et al., 2017). Moreover, several extracellular cues, including growth factors and the ECM, have been shown to modulate AH outflow by altering actin cytoskeletal organization, cell adhesive interactions, contractile characteristics, and stiffness of the trabecular meshwork (Braunger et al., 2015; Vranka et al., 2015; Rao et al., 2017). In addition, the Rho kinase inhibitor and nitric oxide donor induced decrease in IOP, and the use of these agents for treatment of ocular hypertensive primary open-angle glaucoma patients further supports the significance of actin cytoskeletal network, cell adhesive interactions, and contractile characteristics of TM and Schlemm’s canal cells in the modulation of AH outflow (Inoue and Tanihara, 2013; Kawase et al., 2016; Rao et al., 2017; Kopczynski and Heah, 2018; Reina-Torres et al., 2021). While these agents lower IOP effectively, the use of these drugs is associated with multiple adverse effects and less than desirable efficacy for IOP management in glaucoma patients (Terao et al., 2017; Hoy, 2018; Kopczynski and Heah, 2018; Saito et al., 2019). Therefore, there is a need to thoroughly understand the various molecular mechanisms regulating actin cytoskeletal organization, cell-ECM adhesion, contractile characteristics, and mechanotransduction within the TM and Schlemm’s canal to support identification of novel therapeutic targets.

The long-term use of glucocorticoids is recognized to cause elevation of IOP in human patients and in animal models *via* increasing the resistance, barrier, and fibrogenic activities, and by impairing the phagocytic activity of the TM cells (Matsumoto and Johnson, 1997; Clark and Wordinger, 2009; Dibas and Yorio, 2016; Roberti et al., 2020). Moreover, treatment of TM and Schlemm’s canal cells with glucocorticoid has been found to increase cell stiffness, contractile characteristics, cell adhesive interactions, ECM production, and mechanosensitive transcriptional activity (Johnson et al., 1997; Overby et al., 2014; Raghunathan et al., 2015; Bermudez et al., 2017a; Filla et al., 2017; Peng et al., 2018; Yemanyi et al., 2020). In addition, dexamethasone (Dex) treatment of TM cells has been documented to induce the formation of actin cytoskeletal polygonal arrays exhibiting a geodesic architecture, also described as cross-linked actin networks (CLANS) (Bermudez et al., 2017a). Elevated levels of CLANS were also evidenced in the TM cells of glaucoma patients (Filla et al., 2011; Bermudez et al., 2017a). Despite the documented association between elevated levels of CLANS and increased cell and tissue stiffness, mechanosensitive transcriptional activity and fibrogenic activity in the TM (Bermudez et al., 2017a), we have limited understanding of the molecular basis of CLANS formation, and the myriad effects of glucocorticoids in the TM in the context of elevated IOP and increased resistance to AH outflow (Clark and Wordinger, 2009; Dibas and Yorio, 2016; Bermudez et al., 2017a; Roberti et al., 2020).

Moreover, it is becoming increasingly evident that several cell adhesive and certain actin-binding proteins, including actin, shuttle between the cytosolic and nuclear compartments to regulate transcriptional and chromatin remodeling activities (Asp et al., 2002; Benmerah et al., 2003; Wang and Gilmore, 2003; Lundquist et al., 2014; Piccolo et al., 2014; Wang, 2014; Dupont, 2016; Chang et al., 2018; Klages-Mundt et al., 2018; Mahmood et al., 2021). Although several studies have examined the effects of glucocorticoids on the proteome and transcriptome profile of TM cells (Lo et al., 2003; Nehme et al., 2009; Bollinger et al., 2012; Clark et al., 2013; Bermudez et al., 2017b; Shan et al., 2017; Faralli et al., 2019), none of these have focused on characterizing the effects of glucocorticoids either on the cytoskeletome per se or on the nuclear fraction proteome of TM cells. To gain insights into glucocorticoid-induced cellar and molecular changes in the TM, especially changes involving actin cytoskeletal reorganization, cell adhesive interactions, and contractile characteristics, herein, we have utilized proteomics analysis to investigate the effects of Dex on the nuclear protein profile of human TM cells. This study identifies and reveals significant changes not only in the levels of actin interacting and cell adhesive proteins but also in actin-dependent chromatin remodeling proteins and regulators of nuclear actin organization in Dex treated TM cells, offering new molecular insights into glucocorticoid-induced effects in TM and ocular hypertension.

## Materials and Methods

### Human Trabecular Meshwork Cell Culture

Trabecular meshwork primary cells were cultured from human TM tissue isolated from donor corneal rings (ages 18, 20, 22, 38, 41 48, and 63 years, with no known ocular complications), leftover from corneal transplantation surgeries performed at the Duke Ophthalmology Clinical Service, as previously described (Pattabiraman et al., 2014). TM cell culture studies were performed per consensus recommendations described for trabecular meshwork cell isolation, characterization, and culture (Keller et al., 2018). Cells derived from TM tissue were passaged prior to use in experiments between passages 3–6, as described previously (Pattabiraman et al., 2014). TM primary cells were cultured in Dulbecco’s Modified Eagle complete growth medium (DMEM) containing 10% FBS (heat-inactivated fetal bovine serum), PSG (Penicillin (100U/500 ml)-Streptomycin (100 µg/500 ml)-Glutamine (4 mM) at 37°C in an aseptic incubator under 5% CO_2_.

### Dexamethasone Treatment and Preparation of Nuclear Protein Fraction From Trabecular Meshwork Cells

Human TM cells were cultured in 10 cm plastic Petri dishes (Cat. No: 30702115, Eppendorf, Enfield, CT) in the presence of DMEM media as described previously. When cells reached 80–90% confluence, serum content of media was dropped to 5% prior to the addition of dexamethasone (Dex; 0.5 µM) dissolved in ethanol. An equal amount of ethanol (1 µl/ml media) was added to the respective control plates. Medium exchange was performed on alternate days, with Dex being added daily over a period of 7 days. At the end of the treatment period, cells were harvested to isolate cytosolic and nuclear fractions using the Nuclear/Cytosol Extraction Kit (Catalog #: K266 BioVision Inc, CA, United States). The nuclear fraction protein was quantified using a Micro BCA™ protein assay kit (Cat. No: 23235, Thermo Scientific™ Rockford, IL) and used for proteomic and immunoblotting analyses.

For experiments involving either a 24 h or a 5-day Dex treatment, human TM cells were grown to 80–90% confluence in 10 cm dishes. Serum content was dropped to 2% prior to treating cells with 0.5 µM Dex with media being changed on a daily basis. Following Dex treatment, cells were harvested for nuclear extraction, which was performed using a protocol adapted from the ThermoFisher (BioSource: C-070276 1107). In brief, the cells were washed with chilled phosphate-buffered saline (PBS), carefully scraped into 500 μl of a 1x hypotonic buffer (20 mM Tris-HCl, pH 7.4, 10 mM NaCl and 3 mM MgCl_2_) containing protease and phosphatase inhibitors (one tablet each/10 ml buffer, Roche Pharmaceuticals. Basel, Switzerland), transferred into pre-chilled microcentrifuge tubes and pipetted up and down several times, and incubated on ice for 15 min. Twenty-five microliters (25 μl) of 10% NP-40 were added to each sample, followed by vigorous vortexing for 10 s and centrifugation of homogenates for 10 min at 3,000 rpm at 4°C using ThermoFisher Sorvall Legend micro 21R centrifuge. The supernatants were transferred into separate microfuge tubes and saved as cytoplasmic fractions. The pellets were re-suspended in 50 μl of complete cell extraction buffer (10 mM Tris, pH 7.4, 2 mM Na_3_VO_4_, 100 mM NaCl, 1% Triton X-100, 1 mM EDTA, 10% glycerol, 1 mM EGTA, 0.1% SDS, 1 mM NaF, 0.5% deoxycholate, 20 mM Na_4_P_2_O_7_, 1 mM PMSF) containing protease and phosphatase inhibitors, placed on ice for 30 min, and vortexed at 10 min intervals. Resuspended pellet samples were centrifuged at 14,000 x g for 30 min at 4°C, and the supernatants (nuclear protein fraction) were transferred to clean microcentrifuge tubes. Protein was quantified using a Micro BCA™ protein assay kit and used in proteomic and immunoblotting analyses.

### Mass Spectrometry

Sample preparation and LC-MS/MS analysis: Equal amounts of protein (25 µg protein) from control and Dex treated samples were solubilized in 2% sodium dodecyl sulfate, 100 mM Tris-HCl (pH 8.0), reduced with 10 mM dithiothreitol, alkylated with 25 mM iodoacetamide, and subjected to tryptic hydrolysis using the HILIC beads SP3 protocol (ReSyn Biosciences, Gauteng, South Africa) (Hughes et al., 2014). Each digest was dissolved in 12 μl of a 1/2/97% (by volume) trifluoroacetic acid/acetonitrile/water solution, and 3 μl were injected onto a 5 μm, 180 μm × 20 mm Symmetry C18 trap column (Waters Corp. Milford, MA) run using 1% acetonitrile in water for 3 min at 5 μl/min. The analytical separation was next performed using an HSS T3 1.8 μm, 75 μm × 200 mm column (Waters) over 90 min at a flow rate of 0.3 μl/min at 55°C. The 5–30% mobile phase B gradient was used, where phase A was 0.1% formic acid in water and phase B 0.1% formic acid in acetonitrile. Peptides separated by liquid chromatography (LC) were introduced into the Q Exactive HF Orbitrap mass spectrometer (Thermo Fisher Scientific, Waltham, MA) using positive electrospray ionization at 2000 V and capillary temperature of 275°C. Data collection was performed in the data-dependent acquisition (DDA) mode with 120,000 resolution (at m/z 200) for MS1 precursor measurements. The MS1 analysis utilized a scan from 375–1450 m/z with a target AGC value of 1.0e6 ions, the RF lens set at 30%, and a maximum injection time of 50 ms. Advanced peak detection and internal calibration (EIC) were enabled during data acquisition. Peptides were selected for MS/MS using charge state filtering (2–5), monoisotopic peak detection and a dynamic exclusion time of 25 s with a mass tolerance of 10 ppm. MS/MS was performed using higher-energy C-trap dissociation (HCD) with a collision energy of 30 ± 5% with detection in the ion trap using a rapid scanning rate, automatic gain control target value of 5.0e4 ions, maximum injection time of 150 ms, and ion injection for all available parallelizable time enabled.

Protein identification and quantification: For label-free relative protein quantification, raw mass spectral data files were imported into Progenesis QI for Proteomics 4.2 software (Nonlinear Dynamics) for duplicate runs alignment of each preparation and peak area calculations. Peptides were identified using Mascot version 2.6.2 (Matrix Science) for searching human UniProt 2019 reviewed database containing 20,237 entries. Mascot search parameters were 10 ppm mass tolerance for precursor ions; 0.025 Da for fragment-ion mass tolerance; one missed cleavage by trypsin; fixed modification was carbamidomethylation of cysteine; variable modifications were oxidized methionine and Asn/Gln deamidation. Only proteins identified with two or more peptides (protein confidence *p* < 0.05 and false discovery rate <1%) were included in the protein quantification analysis. To account for variations in experimental conditions and amounts of protein material in individual LC-MS/MS runs, the integrated peak area for each identified peptide was corrected using the factors calculated by the automatic Progenesis algorithm utilizing the total intensities for all peaks in each run. Values representing protein amounts were calculated based on a sum of ion intensities for all identified constituent non-conflicting peptides (Reidel et al., 2011).

For protein profiling, we compared relative amounts of proteins confidently identified in control and treated samples. Proteins with at least a two-fold change in abundance and ANOVA P scores less than 0.05 were selected as statistically significant.

### Immunoblotting

Human TM cells grown to 90% confluence in 6-well dishes (Cat. No: 30720113, Eppendorf, Enfield, CT) were treated with 0.5 µM Dex in 2% FBS DMEM or ethanol (control), as described earlier, for either 5 or 7 days. Cells were washed with 1x cold PBS and incubated on ice for 5 minutes with 10% ice-cold trichloracetic acid (TCA) and 0.5M dithiothreitol (DTT). Following several washes with cold deionized water (DI), cells were scraped and transferred into Eppendorf tubes, washed again with cold DI water, and finally washed with diethylether. Precipitates obtained after centrifugation at 16,000xg were suspended in 8 M urea buffer containing 20 mM Tris, 23 mM glycine, and 10 mM DTT saturated in sucrose, protease, and phosphatase inhibitors, as mentioned earlier, and briefly sonicated. Protein concentration was determined using the Micro BCA method, as mentioned previously.

For immunoblotting, equal amounts of nuclear fraction protein (10 µg) from 5- and 7-day Dex treated cells and respective control cells or from whole-cell fractions (40 µg of protein) were mixed with Laemmli sample buffer and separated on 8–12% SDS-PAGE gels, followed by transfer to nitrocellulose membranes, as described previously (Pattabiraman et al., 2014). Membranes were blocked for 2 h at room temperature in Tris-buffered saline (TBS) containing 5% (wt/vol) nonfat dry milk and 0.1% Tween-20 and subsequently probed overnight at 4°C with respective primary antibodies (Supplementary Table S1). Membranes were washed with TBS buffer containing 1% Tween-20 and incubated with appropriate secondary antibodies for 2 h at room temperature. Immunoblots were developed by enhanced chemiluminescence (Thermo Scientific, IL, USA), followed by scanning and analysis using ChemiDoc Touch imaging and Image Lab™ Touch Software (Bio-Rad Laboratories, Hercules, Ca), respectively.

### Immunofluorescence

Human TM cells were grown to ∼70% confluence on gelatin (2%)-coated glass coverslips in complete growth media, washed with phosphate-buffered saline (PBS), and fixed with 4% formaldehyde for 10 min. For determining the Dex effects on the cellular distribution of MICAL-2, SRF and MRTF-A, human TM cells grown on glass coverslips were treated with Dex (0.5 µM) for 7 days as described earlier and fixed with 4% formaldehyde. Cells were subsequently washed with cytoskeletal buffer (10-mM 2-[N-morpholino] ethane sulfonic acid [MES], containing 150-mM NaCl, 5-mM EGTA, 5-mM MgCl_2_, and 5-mM glucose [pH 6.1]), and permeabilized for 10 min with 0.5% Triton X-100 in PBS, and blocked with serum-containing buffer (10% FBS in PBS with 0.02% sodium azide) prior to immunostaining with primary antibodies (Supplementary Table S1). Immunostaining was performed overnight in serum-containing buffer with 0.2% saponin, followed by the appropriate secondary antibodies conjugated with Alexa fluorophores 488 or 568, as described previously (Maddala and Rao, 2020). Cell nuclei were counterstained with Hoechst (Hoechst 33258, Molecular Probes, Eugene, OR). Finally, coverslips were mounted onto glass slides using Shandon Immu-Mount (Thermo-Fisher Scientific) and then imaged using a Nikon Eclipse 90i confocal laser-scanning microscope.

### Cell Viability

To determine whether Dex treatment impacts cell viability or exerts toxic effects under the conditions used in our studies, human TM cultures grown on glass coverslips were treated with Dex (0.5 µM) for 7 days, and control cells were treated with ethanol alone (1 μl/ml media), as described previously. Following treatment, cells were washed with 1X PBS and incubated for 5 min at 37°C with 20 μg/ml of fluorescein diacetate (cell viability stain, Cat. No. F7378, Millipore Sigma, St. Louis, MO) and 0.5 μg/ml propidium iodide (Cat. No: P4170, Millipore Sigma) in serum-free DMEM media. Images were captured under a fluorescent microscope (10X, Zeiss Axioplan 2). A minimum of 10 images were captured at different locations on the coverslip. The number of fluorescence positive cells per unit area were plotted for purposes of quantification.

### siRNA Treatment

Human TM cells grown in 6-well cell culture plates (60–80% confluence), as described previously, were incubated for 20 mins with Opti-MEM (Gibco) containing Lipofectamine^®^ RNA iMAX transfection reagent (ThermoFisher Scientific) and 30 picomol of BRG1 siRNA or scrambled siRNA (Cat. No: sc-29827; sc-37007, Santa Cruz Biotechnology, Dallas, TX). Following siRNA treatment, cell culture media were supplemented with media containing 10% FBS, and incubation continued for 72 h. At the end of the treatment period, cells were harvested by precipitating in 10% TCA buffer for 5 min, followed by washing with deionized water and scraping into urea sample buffer. These samples were sonicated and centrifuged at 800xg for 10 min, with the entire supernatant being saved as a whole fraction. Protein content was determined using the Micro BCA protein assay kit (Thermo Fisher Scientific), as mentioned previously, with 20–40 µg of protein from each sample used for immunoblotting analyses.

To evaluate the effects of BRG1 deficiency on elevated expression of selected proteins by Dex treatment of human TM cells, two sets of cells grown to 60–80% confluence were treated with either BRG1 siRNA or a scrambled siRNA control, as mentioned earlier. After 24 h of treatment, the BRG1 siRNA and control siRNA transfected cultures were treated with ethanol (1 µl//ml media) or Dex (0.5 µM) for two consecutive days prior to harvesting with TCA/Urea sample buffer. Protein fractions were prepared and quantified, and 20–40 µg of protein samples was used for immunoblotting analysis.

### Statistical Analysis

Densitometry analysis of immunoblots was performed using ImageJ software (http://imagej.nih.gov/ij/; provided in the public domain by the National Institutes of Health, Bethesda, MD, United States). Data were normalized to the specified loading controls. Statistical analyses of densitometric data were performed using GraphPad Prism 9 software. Mann–Whitney T-test (one-tailed) was performed to assess the significance of differences between two unpaired and nonparametric variables. To determine the significance between four variables, an ordinary one-way ANOVA with Bonferroni’s multiple comparisons test with a single pooled variance was performed.

## Results

The broad goal of this project was to identify not only significant Dex-induced changes in the protein profile but to also capture the global, time-dependent trends and patterns in protein profile alterations in TM cells, and thus gain insights into the potential feed forward and backward regulatory molecular mechanisms underlying the observed changes. Since the experiments described herein involved different strains of TM cells (obtained from donors differing in age and gender), data for proteins exhibiting at least a two-fold change in expression in response to Dex treatment were grouped across strains, independently of statistical significance. We have, however, clearly identified whether the observed changes in Dex-induced protein expression in human TM cells were statistically significant for each protein identified in the proteomics analyses presented in this manuscript. A small number of proteins was also identified based on a single peptide and clearly marked to indicate whether these proteins exhibited a ≥2-fold change in expression level. Importantly, all data have been made available to readers such that the profile for a given protein of interest can be screened in detail (MassIVE MSV000088981; dataset license: CC0 1.0 Universal (CC0 1.0).

### Alterations in the Nuclear Protein Profile of Human Trabecular Meshwork Cells Treated With Dexamethasone for Seven Days

**FIGURE 1:**
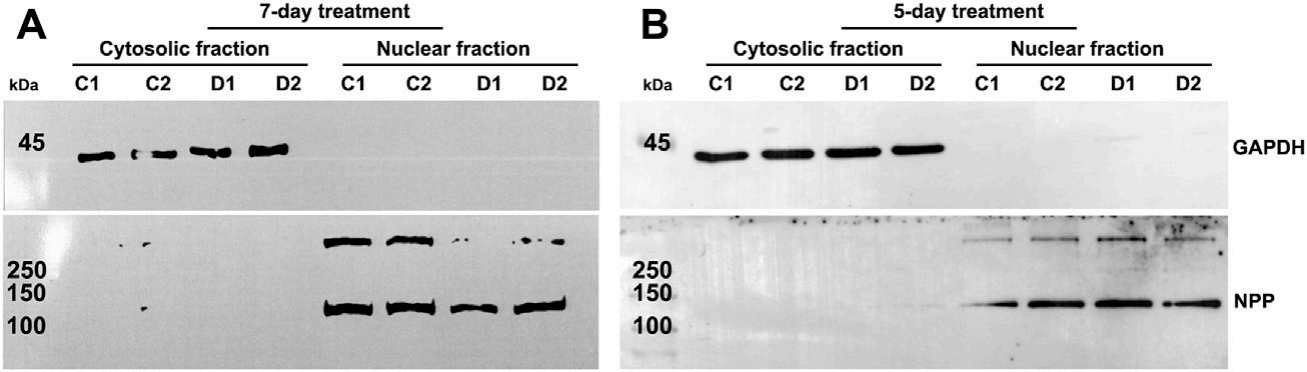
Confirmation of purity of nuclear protein fractions isolated from human trabecular meshwork (TM) cells. **(A,B)** depict immunoblotting results for glyceraldehyde 3-phosphate dehydrogenase (GAPDH) and nuclear pore protein (NPP) in cytosolic and nuclear protein fractions, respectively, isolated from human TM cells treated with dexamethasone (Dex) for a period of 5- or 7-days. The nuclear protein fractions show strong staining for NPP but no immunopositive bands for GAPDH, confirming the purity of the nuclear extracts. C1 and C2 and D1 and D2 represent control replicates 1 and 2, and Dex treated replicates 1 and 2, respectively. The nuclear protein fractions from 5- and 7-day Dex treated TM cells were isolated using the commercial kit and in house procedures, respectively, as described in the Methods section.

In an initial experiment, we evaluated one strain of human TM cells in duplicate (same strain of TM cells plated in two separate 10 cm plastic dishes). Cells were treated either with 0.5 µM Dex or vehicle (ethanol, 1 μl/ml media) alone for 7 days with daily addition of Dex or vehicle and change of culture medium containing 5% FBS on alternate days. Following treatment, nuclear protein fractions were prepared as described in the Methods section. The purity of nuclear fractions was evaluated by the presence of GAPDH contamination and confirming the presence of nuclear pore protein (NPP), a nuclear protein marker, by immunoblot analysis. As shown in Figure 1A, nuclear protein fraction samples derived from TM cells had negligible GAPDH contamination and were enriched for NPP. In addition, under the afore-described conditions, as has been shown previously (Bermudez et al., 2017a), Dex induced actin stress fibers and CLANS formation in human TM cells compared to control cells (Supplementary Figure S1).

Analysis of nuclear protein fractions from duplicate cultures of Dex treated human TM cells derived from the same strain identified 73 and 66 proteins in replicates 1 and 2, respectively, whose expression levels were significantly increased (by a minimum of 2-fold) compared to the respective controls (Supplementary Table S2). Of the significantly upregulated proteins, 43 proteins were detected in both Dex treated cultures (duplicates) from the same human TM strain. The increase (≥2-fold) in levels for eleven of the upregulated proteins was not statistically significant relative to untreated controls (Supplementary Table S2), with seven of these being identified in nuclear fractions of both Dex treated duplicate samples (Supplementary Table S2). Keratins detected only in one of the duplicate samples were not listed. This analysis of nuclear protein fractions from duplicate cultures of Dex treated human TM cells derived from the same strain also identified 54 and 43 proteins in replicates 1 and 2, respectively, whose levels were significantly (*p* < 0.05) decreased by a minimum of 2-fold (Supplementary Table S3) compared to controls. Of these, 23 proteins were identified in both Dex treated duplicates. Eight of the downregulated proteins exhibited a ≥ 2-fold decrease, but the difference was not found to be statistically significant compared to controls. There was one protein which was common between the duplicates. Overall, these initial results confirmed very good consistency between duplicate analyses of the same strain of TM cells regarding the effects of Dex on the nuclear protein profile.

In these initial analyses, we also performed proteomics analyses of cytosolic proteins from the duplicate Dex treated and control cultures derived from the same strain of human TM cells. Some of the proteins which were found to be elevated in the nuclear fraction under Dex treatment were also increased in the cytosolic fraction, indicating that Dex treatment increases the total levels for some of the described proteins (*see data repository files*). However, we have not analyzed the cytosolic fractions for all other samples described in this study.

A GO (Gene Ontology) terms/enrichment analysis was then performed for proteins whose levels were altered in response to Dex treatment. For this, we used gene identification accession numbers for the identified proteins. Proteins identified in both replicates (common ones) of the same strain of TM cells and those identified in the individual replicates were included in this analysis to gain broader insight into what biological processes and molecular functions are involved in the Dex-induced effects on TM cells. Interestingly, the subset exhibiting Dex-induced increases in expression level was enriched for proteins involved in actin cytoskeletal organization, including actin cytoskeletal bundling, stress fiber assembly, regulation of actin filament organization, endocytosis, cell adhesion, signal transduction, cellular localization, and vesicle transport. These findings were consistent for proteins identified in both duplicates (Supplementary Figure S2A) and those detected in the individual replicates from the same strain of human TM cells (Supplementary Figure S2B,C). In contrast to the enrichment terms analysis for proteins upregulated by Dex, the downregulated subset was found to be consistently enriched for proteins involved in collagen fibril organization, SRP (signal recognition particle)-dependent cotranslational protein targeting to membrane, viral transcription, and in catabolic and metabolic processes of RNA, nucleic acid, heterocycle and nitrogen compounds in both duplicate samples (Supplementary Figure S3A,B). Supplementary Figure S3C shows the results of enrichment analysis of downregulated genes identified in nuclear fractions from duplicate Dex treated human TM cultures derived from the same strain, and the findings were largely similar to the results found in the individual samples.

The previously described analyses were expanded to two additional human TM cell strains, one of which was analyzed in duplicate and the other was analyzed as a single replicate (with Dex and vehicle). A total of three independent strains of human TM cells were thus utilized to generate five technical replicate analyses for the 7-day time point of Dex treatment. Based on these analyses, proteins whose expression levels were modulated in three or more of the five samples per treatment were then compared to their respective controls. Table 1 lists the proteins exhibiting upregulated expression by ≥ 2-fold in response to Dex treatment. Levels of the following proteins were consistently and significantly increased in response to Dex treatment: CNN3, ESP8, FERM2 (*FERMT2*), GPC4, ITAV (*ITGAV*), ITB5 (*ITGB5*), LIMC1 (*LIMCH1*), LTBP2, MYOC, PLPP3, PPME1, SEPT11 (*SEPTIN11*), SI1LI (*SIPA1L1*), SRBS1 (*SORBS1;* CAP/Ponsin), SRBS2 (*SORBS2;* ArgBP2), STOM, SYNJ1, and TENS1. In those cases where the protein and the corresponding gene ID differ, gene IDs (in italics) are provided in parentheses.

**TABLE 1:** Dexamethasone treatment (for 7 days) induced increase in the levels of specific proteins in the nuclear fraction derived from three or more human TM samples.

Accession	Description	Significant
ACOT9	Acyl-coenzyme A thioesterase 9, mitochondrial	
APOB	Apolipoprotein B-100	**∗**
CAND1	Cullin-associated NEDD8-dissociated protein 1	**∗**
CAVN2	Caveolae-associated protein 2	
CNN2	Calponin-2	
CNN3	Calponin-3	**∗**
CNTN1	Contactin-1	
CO8A1	Collagen alpha-1(VIII) chain	**∗**
CP1B1	Cytochrome P450 1B1	**∗**
DESP	Desmoplakin	
DHC24	Delta (24)-sterol reductase	
DPYL2	Dihydropyrimidinase-related protein 2	
EPS8	Epidermal growth factor receptor kinase substrate 8	**∗**
FBN2	Fibrillin-2	
FERM2	Fermitin family homolog 2	**∗**
FILA2	Filaggrin-2	
FLNB	Filamin-B	**∗**
G6PI	Glucose-6-phosphate isomerase	**∗**
GPC4	Glypican-4	**∗**
HTRA1	Serine protease HTRA1	**∗**
ITAV	Integrin alpha-V	**∗**
ITB5	Integrin beta-5	**∗**
K1C9	Keratin, type I cytoskeletal 9	**∗**
KCD12	BTB/POZ domain-containing protein KCTD12	**∗**
LDHA	L-lactate dehydrogenase A chain	**∗**
LIMC1	LIM and calponin homology domains-containing protein 1	**∗**
LTBP2	Latent-transforming growth factor beta-binding protein 2	
MFGM	Lactadherin	**∗**
MYO6	Unconventional myosin-VI	
MYOC	Myocilin	**∗**
NEP	Neprilysin	**∗**
OCTC	Peroxisomal carnitine O-octanoyltransferase	
PARVA	Alpha-parvin	
PGS2	Decorin	
PLPP3	Phospholipid phosphatase 3	**∗**
PPME1	Protein phosphatase methylesterase 1	**∗**
PYGB	Glycogen phosphorylase, brain form	**∗**
Sep11	Septin-11	**∗**
SI1L1	Signal-induced proliferation-associated 1-like protein 1	**∗**
SNX9	Sorting nexin-9	
SRBS1	Sorbin and SH3 domain-containing protein 1 (CAP/Ponsin)	**∗**
SRBS2	Sorbin and SH3 domain-containing protein 2 (ArgBP2)	**∗**
STOM	Erythrocyte band 7 integral membrane protein	**∗**
SYNJ1	Synaptojanin-1	**∗**
TENS1	Tensin-1	**∗**
TIMP3	Metalloproteinase inhibitor 3	
UGPA	UTP-glucose-1-phosphate uridylyltransferase	**∗**
VINEX	Vinexin	

Footnote: Proteins whose levels are elevated by ≥ 2-fold in response to Dex treatment are listed. “*” indicates a significant (*p* < 0.05) increase in the level of identified protein in nuclear extracts from Dex treated human TM cells relative to control human TM cells.

**FIGURE 2:**
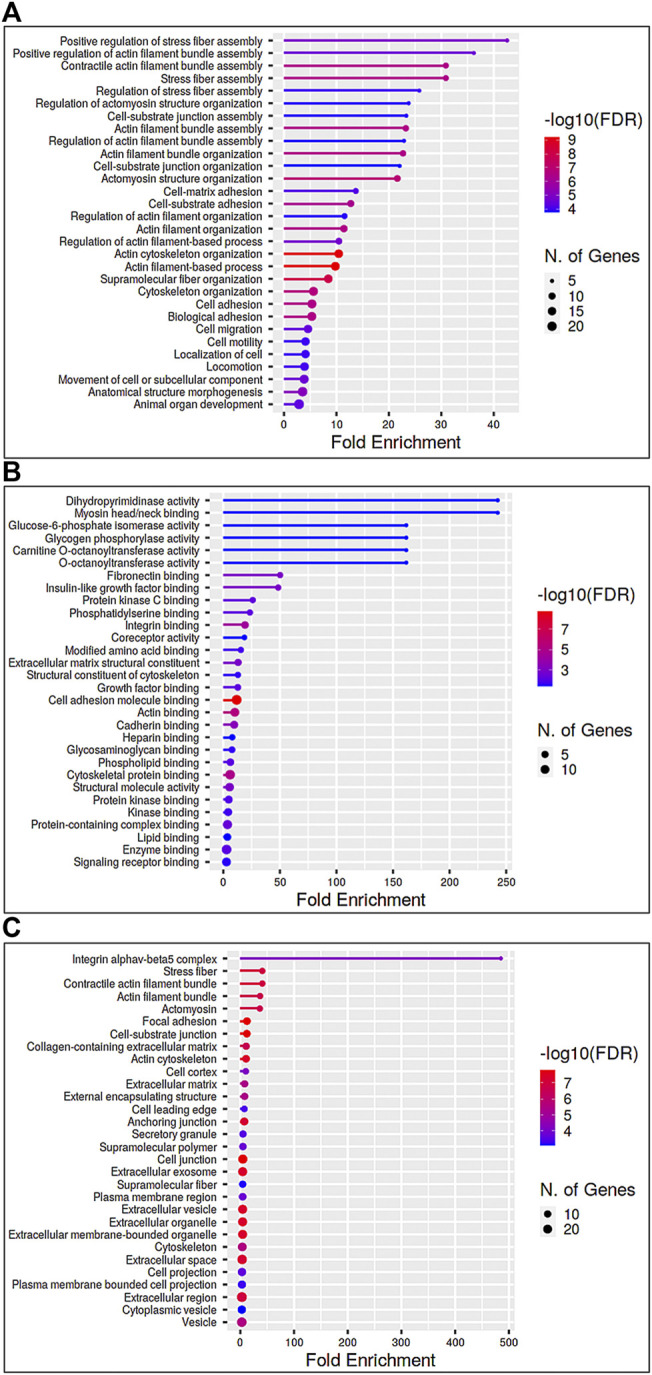
Gene Ontology enrichment analysis of proteins elevated in the nuclear protein fraction of Dex treated (for 7 days) human TM cells. Panels **(A–C)** depict Gene Ontology (GO) enrichment analyses for biological process, molecular function, and cellular components, respectively (using ShinyGO v0.75 online), of proteins exhibiting a ≥2-fold increase in three or more samples of TM cells treated for 7 days with Dex. Analysis was performed using a P-value cutoff (FDR) of 0.05, and results are shown for the top 30 pathways identified *via* GO analysis.

As described earlier, analysis of GO terms enrichment for Dex upregulated proteins found in a minimum of three samples, and representing a minimum of two strains of human TM cells (see Figures 2A–C) detected protein enrichment for biological process, molecular function, and cellular components, respectively. Interestingly, all three categories revealed enrichment for proteins involved in actin filament bundling, organization, contraction, stress fiber assembly, myosin, cell adhesion, cadherin, supramolecular fiber organization, vesicle-mediated transport, ECM, extracellular vesicle and others. Collectively, these findings reveal that Dex treatment upregulates the expression of proteins involved predominantly in actin cytoskeletal assembly, bundling, organization, contraction, cell adhesion, and vesicle transport. In contrast, proteins downregulated by Dex included those related to the catabolic and metabolic process of nucleic acids, peptides and heterocycle, viral gene expression, fibrinolysis, protein targeting to membrane, and general metabolism (Supplementary Table S4; Supplementary Figure S4).

**FIGURE 3:**
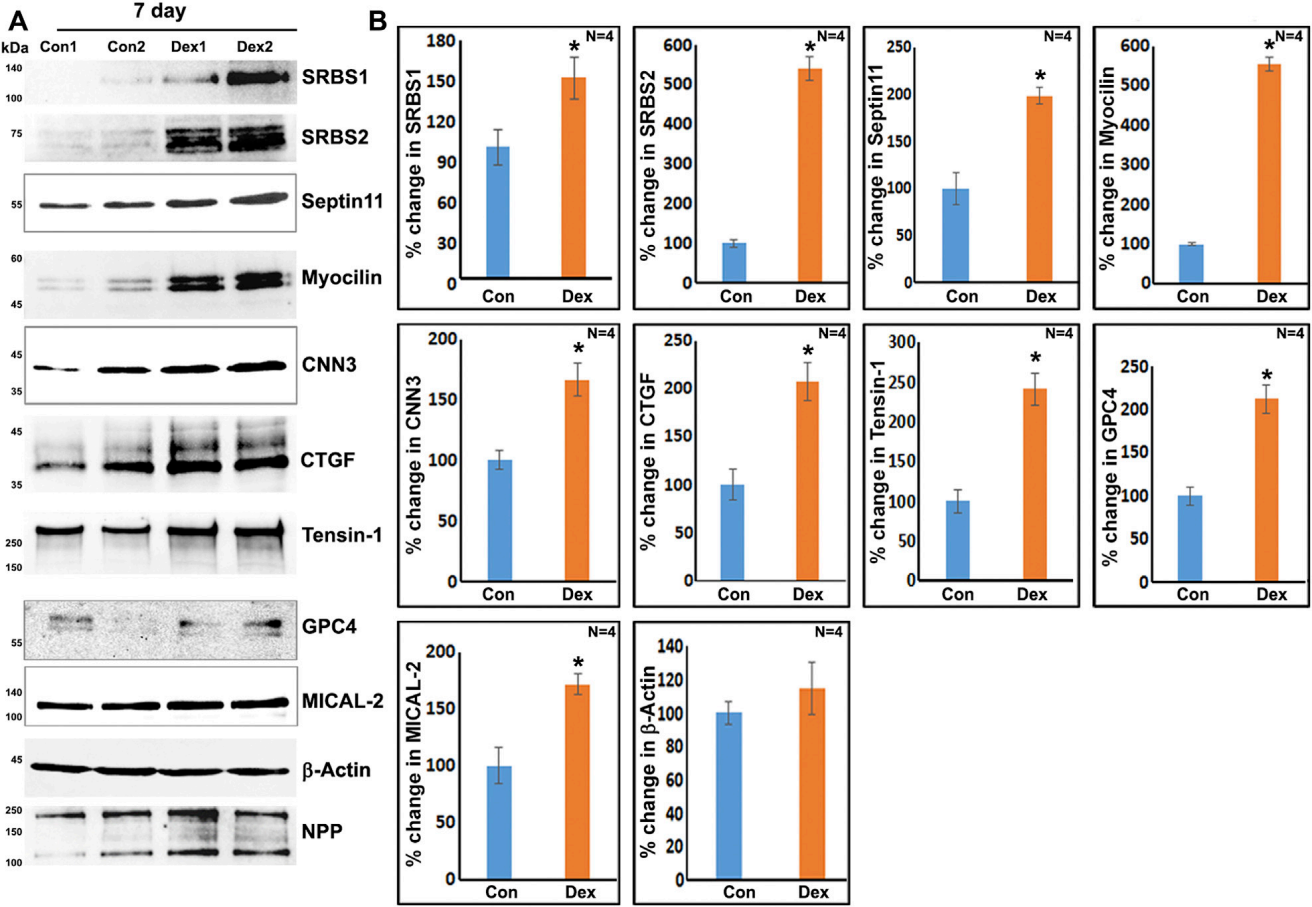
Immunoblotting analysis based confirmation of elevated levels for selected proteins in the nuclear protein fraction derived from the 7-day Dex-treated human TM cells. **(A,B)** show the representative immunoblots for the indicated proteins and quantitative changes in the levels of these proteins (histograms), respectively, in the nuclear protein fraction isolated from the TM cells treated with Dex for 7 days. ∗*p* < 0.05. *n* = sample number. Immunoblotted proteins were normalized to the nuclear pore protein (NPP).

**FIGURE 4:**
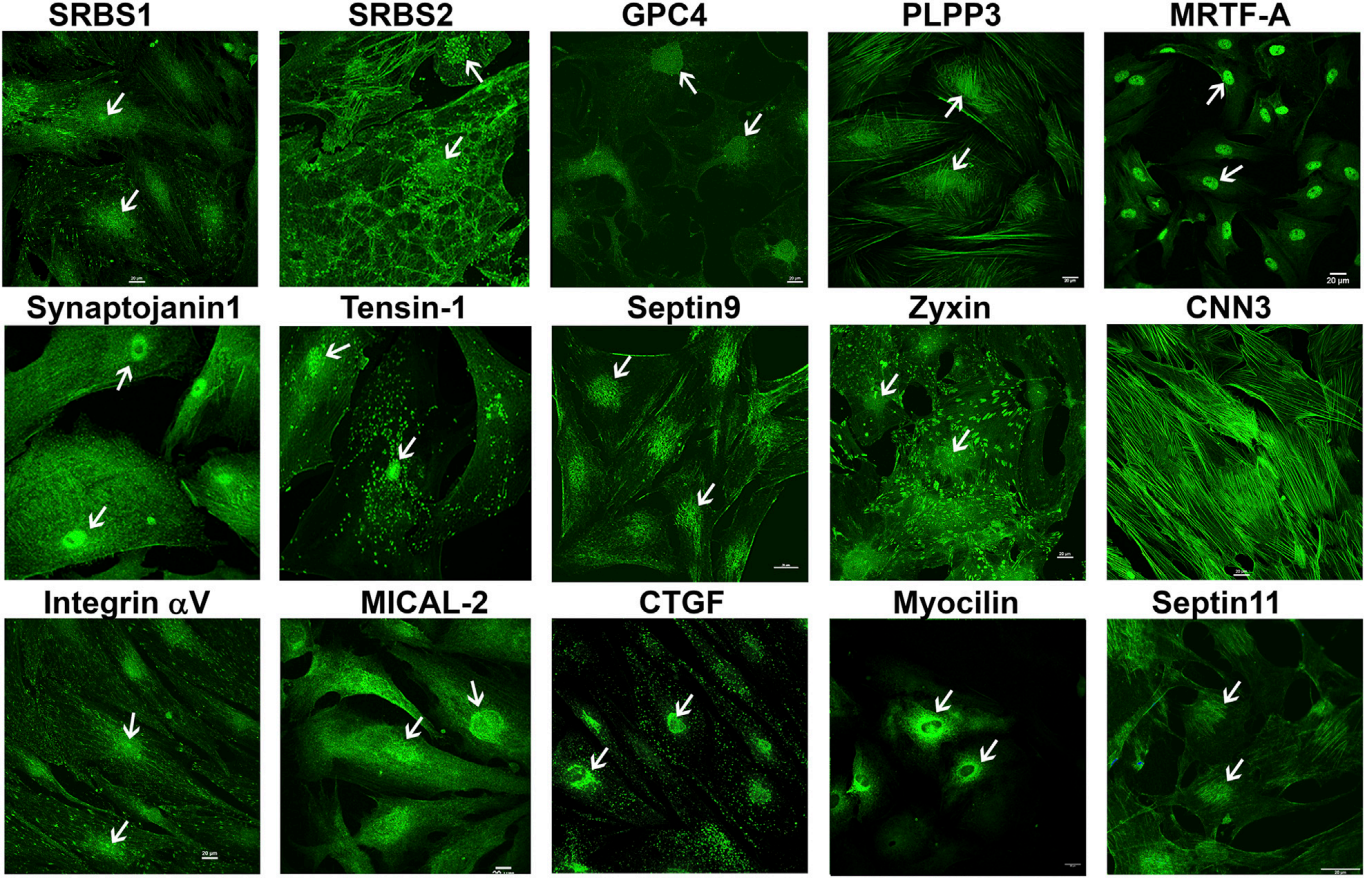
Immunofluorescence based distribution pattern of proteins whose levels were increased in the nuclear protein fraction of human TM cells treated with Dex. Several of the indicated proteins were detected in both the cytosol and nucleus of human TM cells under untreated condition. Arrows indicate the cell nucleus. Scale bars indicate magnification.

**FIGURE 5:**
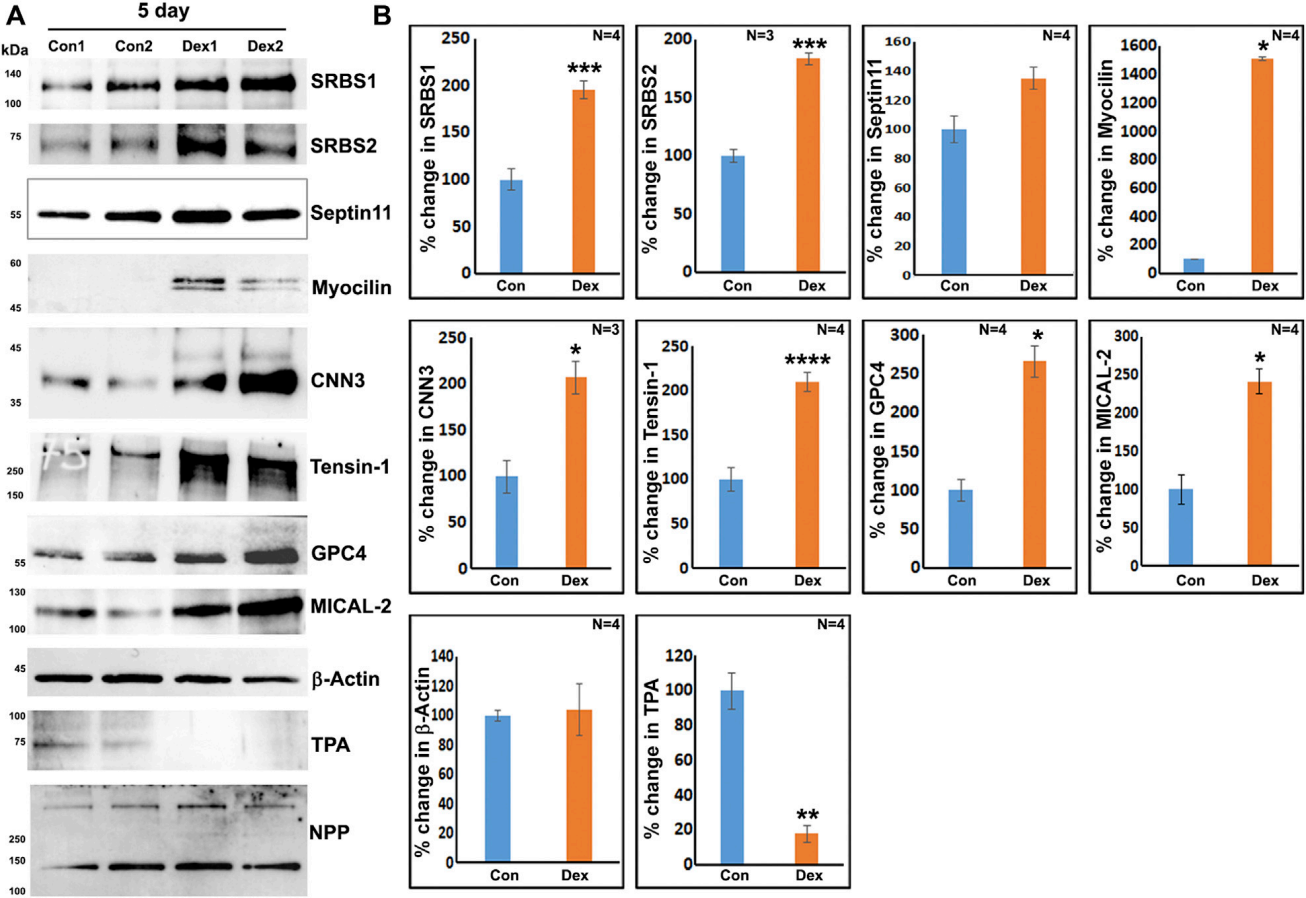
Immunoblotting analysis based confirmation of elevated levels of selected proteins in the nuclear protein fraction derived from the 5-day Dex-treated human TM cells. **(A,B)** show the representative immunoblots for the indicated proteins and quantitative changes in levels of these proteins (histograms), respectively, in the nuclear protein fraction isolated from TM cells treated with Dex for 5 days. *p < 0.05; **p < 0.01; ***p < 0.001; ****p < 0.0001. *n* = sample number. Immunoblotted proteins were normalized to the nuclear pore protein (NPP).

To obtain further confirmation of the findings from proteomics analysis, we performed immunoblotting analyses for selected proteins. These analyses used a separate set of nuclear fractions prepared from Dex treated and control human TM cells. As shown in Figures 3A and B, significant increases were noted in levels of the indicated proteins in nuclear fractions of Dex treated TM cells relative to control cells. Figure 4 shows the distribution profile of some of the Dex-stimulated, differentially expressed proteins in human TM cells (based on immunofluorescence analysis). Some of these proteins reveal both a cytosolic and nuclear distribution, while others are distributed discretely to the nucleus under unstimulated conditions. Levels of the nuclear actin depolymerizing protein involved in activation of SRF/MRTF-A mediated transcriptional activity (Lundquist et al., 2014), MICAL2, were significantly increased in two of the samples (based on proteomics analysis), and a finding was confirmed independently by immunoblotting analysis of nuclear fractions from Dex treated human TM cells (Figures 3 and 5). β-Actin was also readily detected in the nuclear fraction samples of TM cells by immunoblotting analysis (Figures 3 and 5).

### Time-Dependence of Dexamethasone-Induced Effects on the Nuclear Protein Profile of Human Trabecular Meshwork Cells

To characterize the global, time-dependent trends and patterns in protein profile alterations induced by Dex treatment of human TM cells, we used two independent human TM strains to generate a total of six technical replicates. These TM cell strains were treated with 0.5 µM Dex over a 5-day period, with daily changes of Dex-containing media. As noted in the previous (7-day Dex treatment) experiments, consistent observations were noted regarding the effects of Dex on the nuclear protein profiles across technical replicates of the same strain of TM cells. Although a slightly different protocol (in house protocol described in the Methods section) was used for isolation of the nuclear protein fraction from 5-day Dex treated samples, the purity of this fraction was similar to that of the 7-day Dex treated samples (Figure 1B).

Supplementary Table S5 lists proteins that exhibited at least two-fold increases in three or more samples derived from two strains of TM cells. Mass spectrometry–based detection of increased levels of nuclear proteins in 5-day Dex treated human TM cells was independently confirmed by immunoblotting analysis of nuclear protein fractions. Figure 5 shows the increased levels of identified proteins in nuclear protein fractions derived from the 5-day Dex treated samples. We also confirmed the decreased levels of one of the proteins (tissue plasminogen activator) in 5-day Dex treated TM cell samples (Figure 5). GO term enrichment analyses of Dex upregulated proteins in nuclear fractions of 5-day treated human TM cells revealed not only cytoskeletal and cell adhesion proteins but also proteins involved in chromatin remodeling, RNA splicing and processing, nuclear transport, intracellular protein transport, protein localization, and cellular components biogenesis (Supplementary Figure S5). These findings opened up an interesting insight into the possible molecular mechanisms involved in Dex-mediated transcriptional changes. Interestingly, there were significant increases in the levels of BRG1 (Brahma related gene-1), MECP2 and MTA1 in Dex treated cells (Supplementary Table S5). BRG1, an actin-binding, chromatin remodeling protein is well recognized to regulate glucocorticoid-induced gene expression (Fryer and Archer, 1998; Hoffman et al., 2018; Mahmood et al., 2021). It is a catalytic subunit of the SWI/SNF multi-protein chromatin regulatory complex (Khavari et al., 1993; Wilson and Roberts, 2011; Hodges et al., 2016) and is also involved in regulating the expression of actin cytoskeletal proteins through mechanisms including activation of SRF/MRTF-A dependent transcription (Zhang et al., 2007; Zhou et al., 2009). This aspect is discussed further in the Results section.

**FIGURE 6:**
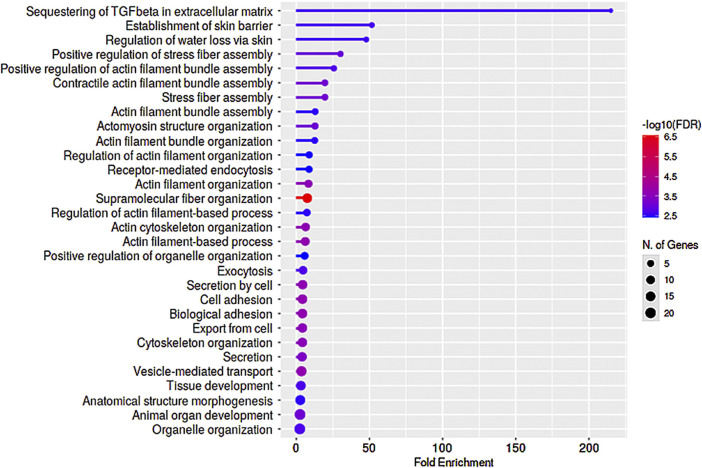
GO enrichment analysis of proteins exhibiting upregulated levels in nuclear protein fractions of human TM cells treated with Dex for 5- and 7-day periods. Several proteins exhibited similar trends (≥2-fold increase) in changes in levels between the 5- and 7-day Dex treated samples, and enrichment analysis for Biological process identified preferential upregulation of proteins involved in actin stress fiber formation, bundling and organization, cell adhesion, and vesicle transport. This analysis was performed using a P-value cutoff (FDR) of 0.05, and results are shown for the top 30 pathways.

We also assessed commonalities in protein profile alterations (≥2-fold increase) across the 5- and 7-day Dex treated TM cells (Table 2). Some examples identified in this analysis include CCN2 (CTGF), CNN3, FBN1, FERM2 (*FERMT2*), FILA2 (*FLG2*), GPC4, HIP1, LIMC1 (*LIMCH1*), LTBP2, PCD10 (*PCDH10*), SEPT7 (*SEPTIN7*), SRBS2 (*SORBS2*; ArgBP2), STOM, SYNJ1, TENS1, VINEX (*SORBS3;* Vinexin), and ZYX. Analysis of GO terms enrichment for the upregulated proteins which are common in 5- and 7-day Dex treated TM cells revealed proteins involved in actin cytoskeletal bundling, organization, stress fiber organization, actomyosin organization, cell adhesion, vesicle transport, exocytosis, endocytosis, and TGF-beta signaling (Figure 6). These findings confirm that upregulation of expression for some of the cytoskeletal, cell adhesion, and vesicle transport proteins detected in 7-day Dex treated human TM cells is initiated at an earlier time point in the treatment schedule. The changes in nuclear protein levels by mass spectrometric analyses as described above were also confirmed by immunoblotting analysis of nuclear fractions from 5-day Dex treated human TM cells and some of their distribution analysis by immunofluorescence (Figures 4 and 5).

**TABLE 2:** Proteins whose levels are elevated by ≥ 2-fold in two or more nuclear fraction samples derived from Dex treated human TM cells at both the 5- and 7-day exposure time points.

Accession	Description
AKAP2	A-kinase anchor protein 2
AP2A2	AP-2 complex subunit alpha-2
APOB	Apolipoprotein B-100
ASAP1	Arf-GAP with SH3 domain, ANK repeat and PH domain-containing protein 1
BCKD	[3-methyl-2-oxobutanoate dehydrogenase [lipoamide]] kinase, mitochondrial
CCN2	CCN family member 2 (CTGF)
CD59	CD59 glycoprotein
CNN3	Calponin-3
CNTN1	Contactin-1
COBA1	Collagen alpha-1 (XI) chain
COTL1	Coactosin-like protein
CP1B1	Cytochrome P450 1B1
CPSF3	Cleavage and polyadenylation specificity factor subunit 3
DESP	Desmoplakin
DEST	Destrin
DHC24	Delta (24)-sterol reductase
DHE3	Glutamate dehydrogenase 1, mitochondrial
DPYL2	Dihydropyrimidinase-related protein 2
EPS15	Epidermal growth factor receptor substrate 15
FBN1	Fibrillin-1
FBN2	Fibrillin-2
FERM2	Fermitin family homolog 2
FHL2	Four and a half LIM domains protein 2
FILA2	Filaggrin-2
FLNB	Filamin-B
GBG12	Guanine nucleotide-binding protein G(I)/G(S)/G(O) subunit gamma-12
GPC4	Glypican-4
HIP1	Huntingtin-interacting protein 1
HORN	Hornerin
ITM2B	Integral membrane protein 2B
ITSN1	Intersectin-1
K1C9	Keratin, type I cytoskeletal 9
K2C1	Keratin, type II cytoskeletal 1
LDHA	L-lactate dehydrogenase A chain
LIMC1	LIM and calponin homology domains-containing protein 1
LTBP2	Latent-transforming growth factor beta-binding protein 2
MFGM	Lactadherin
NEXN	Nexilin
PCD10	Protocadherin-10
PLPP3	Phospholipid phosphatase 3
PPME1	Protein phosphatase methylesterase 1
RAB2A	Ras-related protein Rab-2A
RN168	E3 ubiquitin-protein ligase RNF168
SEPT2	Septin-2
SEPT7	Septin-7
SRBS2	Sorbin and SH3 domain-containing protein 2 (ArgBP2)
STOM	Erythrocyte band 7 integral membrane protein
SYNJ1	Synaptojanin-1
TENS1	Tensin-1
TIMP3	Metalloproteinase inhibitor 3
VINEX	Vinexin
ZN638	Zinc finger protein 638
ZYX	Zyxin

Footnote: Proteins exhibiting increased levels (by ≥ 2-fold) in response to Dex treatment in two or more samples of human TM cells representing both the 5- and 7-day treatment periods are listed.

In addition, we tested the short-term (24 h) effects of Dex on the nuclear protein fraction profile of human TM cells. One strain of human TM cells was analyzed in duplicate for this purpose, which identified a ≥2-fold increase in expression of the following proteins, which are also increased in 7-day Dex treated samples: EHD4, GPC4, ITB5 (*ITGB5*), LTBP2, PCD10 (*PCDH10*), SEP11 (*SEPTIN11*), UGPA, and ZYX. Several of these proteins were also increased in 5-day Dex treated TM cells. In addition, the levels of SMARCD2 (Brg1-associated factor), ENAH (regulator of actin polymerization), HYOU1 (hypoxia up-regulated protein-1), TAGLN2 (Transgelin-2), LMOD1 (Lieomodin-1), LAMP1 (lysosome-associated membrane glycoprotein 1), KIF5B (Kinesin-1 heavy chain), and NOLC1 (nucleolar and coiled-body phosphoprotein 1) were increased by ≥ 2 fold in Dex treated TM cells. Table 3 lists proteins exhibiting elevated or decreased levels in 24 h, 5- and 7-day Dex treated samples. The expression level of the following proteins was found to be elevated at all three intervals (24 h, 5- and 7-day) of Dex treatment: GPC4, LTBP2, PCD10 (*PCDH10*), and ZYX. There were also two proteins, namely, NFM (*NEFM*; neurofilament medium polypeptide) and TPA (*PLAT*; tissue plasminogen activator), whose levels were decreased across all three intervals of Dex treatment.

**TABLE 3:** Proteins whose levels are changed by ≥ 2-fold in two or more nuclear fraction samples derived from human TM cells treated with Dex for 24 h, 5days, and 7 days.

Accession	Description	Enriched or depleted
GPC4	Glypican-4	**↑**
LTBP2	Latent-transforming growth factor beta-binding protein 2	**↑**
PCD10	Protocadherin-10	**↑**
ZYX	Zyxin	**↑**
NFM	Neurofilament medium polypeptide	**↓**
TPA	Tissue-type plasminogen activator	**↓**

Footnote: Proteins whose levels were elevated by two or more folds in two or more samples of 1-, 5-, and 7-day Dex treated TM cells are listed. Arrows pointing up and down reflect an increase and decrease in expression level, respectively.

### BRG1 Regulated Gene Expression in Trabecular Meshwork Cells

**FIGURE 7:**
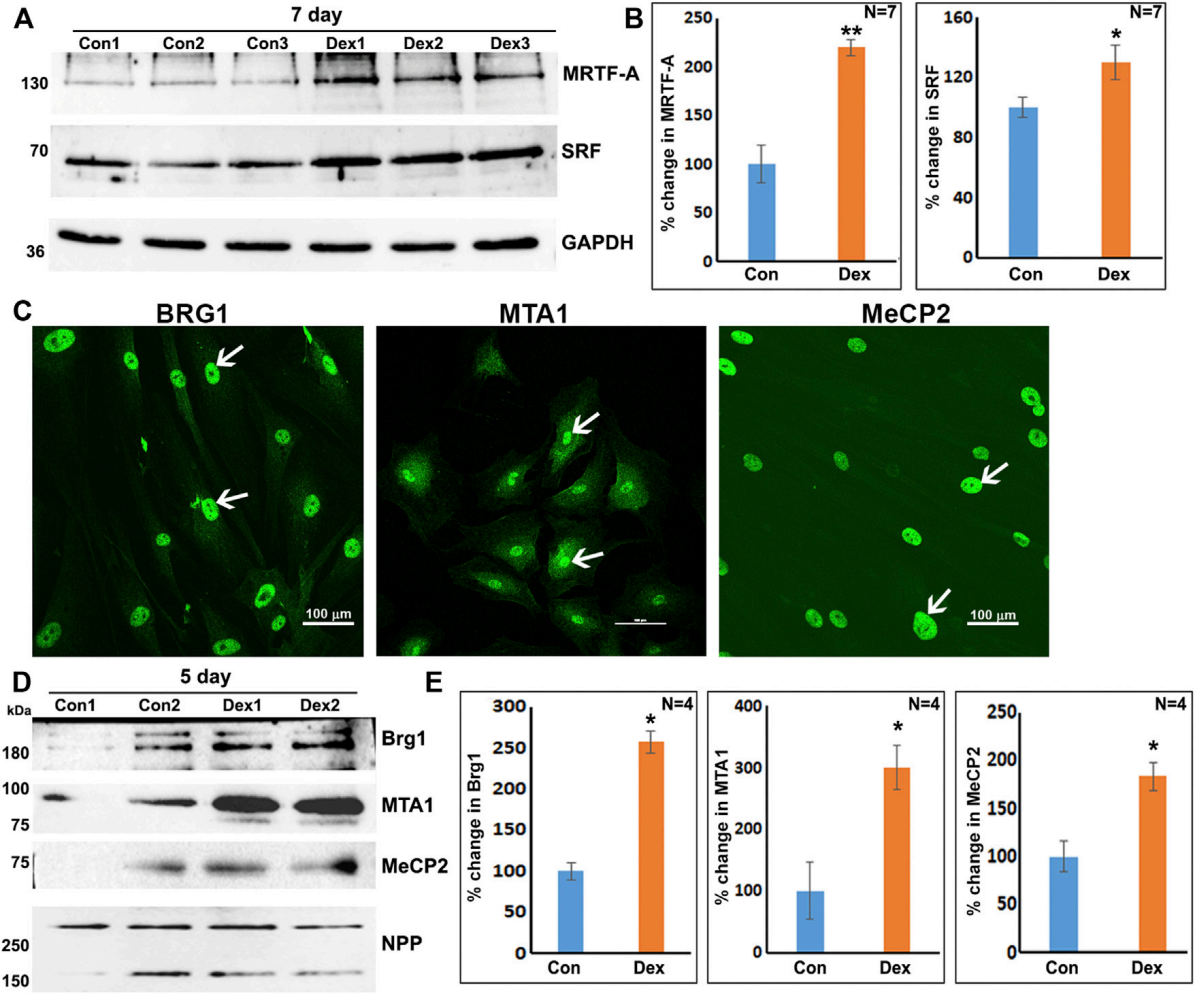
Increased levels of transcriptional (SRF, MRTF-A) and chromatin regulators (BRG1, MTA1, and MeCP2) in Dex treated human TM cells. **(A,B)** Total cell lysates derived from Dex treated (7 days) TM cells showed a significant increase in the levels of SRF and MRTF-A relative to controls. **(C)** Nuclear distribution of BRG1, MTA1, and MeCP2 in human TM cells. **(D,E)** Increased levels of BRG1, MTA1, and MeCP2 proteins in the nuclear protein fraction of Dex treated (5 days) TM cells. *p < 0.05; **p < 0.01. Scale bars indicate image magnification.

Based on the results of proteomics and GO enrichment analysis, it is evident that Dex treatment of human TM cells for 7 days stimulates an increase in the levels of proteins involved predominantly in actin cytoskeletal organization, cell contraction, cell adhesion, and vesicle transport. In contrast, human TM cells treated with Dex for 5 days exhibited increased levels of BRG1, MTA1, and MECP2, which are chromatin regulatory proteins (Xue et al., 1998; Chahrour et al., 2008; Hodges et al., 2016), in addition to some of the proteins upregulated in nuclear fractions of 7-day Dex treated human TM cells. In addition, increases were also detected in the levels of MICAL2 and MICAL2-like proteins in nuclear fractions from 5 to 7 day Dex treated human TM cells, and an increase in BRG1 (in one of the replicates) and BRG1 associated factor (SMARCD2; in both the replicates) levels in the 24 h Dex treated samples. These findings suggest activation of specific chromatin-dependent and transcriptional mechanisms in Dex treated TM cells, which might then be involved in the observed changes in expression of key effector/client proteins identified in our study. Interestingly, both BRG1 and MICAL-2 stimulate SRF/MRFT-A transcriptional activity, considered a master regulator of expression of actin cytoskeletal and contractile proteins in various cell types (Small et al., 2010; Velasquez et al., 2013; Miralles et al., 2003). Moreover, Dex treatment of TM cells for 7 days led to significant increases in the levels of both SRF (serum response factor) and MRTF-A (myocardin related transcription factor A) in total cell lysates as compared to controls (Figure 7A). In addition, the nuclear localization of MICAL2, MRTF-A and SRF was found to be relatively higher in Dex treated human TM cells compared to control cells (Supplementary Figure S6). Interestingly, actin-dependent BRG1 has been shown to play a crucial role in glucocorticoid-induced gene expression in various cell types (Fryer and Archer, 1998; Hoffman et al., 2018). We, therefore, attempted to understand the role of BRG1 in Dex-induced changes in nuclear protein levels of human TM cells.

**FIGURE 8:**
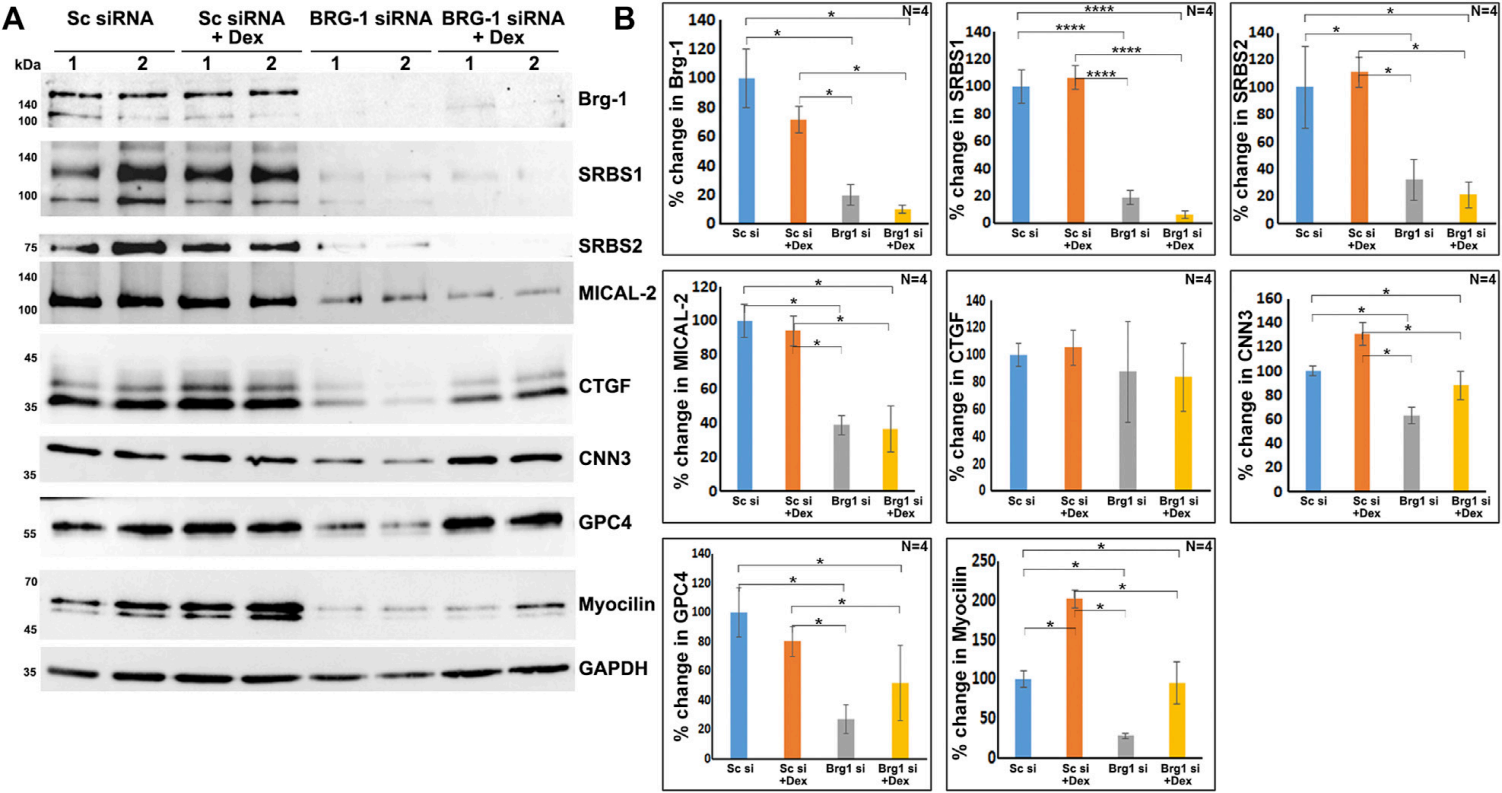
BRG1 deficiency decreases the levels of various proteins in TM cells under both basal and Dex stimulated conditions. **(A,B)** BRG1 siRNA and a corresponding scrambled siRNA control were used to determine the role of the chromatin remodeling protein BRG1 in the expression of the indicated proteins in TM cells. Cells were initially treated with siRNA for 24 h prior to the addition of Dex or vehicle and continuation of incubation for another 48 h. Cell lysates were prepared for immunoblotting based quantification of the proteins of interest. As shown in the figure, treatment with BRG1 siRNA significantly reduced the levels of BRG1 and other indicated proteins in TM cells under both normal and Dex treated conditions. *p < 0.05; ****p < 0.0001. Sc si: Scrambled siRNA; Brg1 si: BRG1 siRNA. Lanes 1 and 2 indicate replicate samples.

As shown in Figure 7, BRG1, MTA1, and MECP2 are distributed predominantly to the nucleus in human TM cells. Immunoblotting analysis confirms the presence of these proteins in the nuclear fraction, and increased expression levels of BRG1, MTA1, and MECP2 in Dex treated human TM cells. Using a siRNA approach, we evaluated whether BRG1 deficiency impacts the ability of Dex to induce an increased expression of nuclear proteins in human TM cells. Treatment of human TM cells with a BRG1 specific siRNA resulted in significantly decreasing the levels of BRG1 in both Dex treated and controls (Figure 8). BRG1 deficiency decreased the Dex stimulated increase in myocilin, SRBS1(*SORBS1*; CAP/Ponsin), SRBS2 (*SORBS2*; ArgBP2), and MICAL2 protein levels in human TM cells, as well the basal level of expression of these proteins in Dex untreated cells, relative to control cells treated with a scrambled siRNA control (Figure 8). CNN3, CTGF, and GPC4 protein levels were decreased in TM cells treated with BRG1 siRNA alone; however expression levels of these proteins between Dex treated BRG1 deficient cells and Dex treated scrambled siRNA controls were found to be marginally different (Figure 8). In experiments evaluating the effects of BRG1 siRNA, cells were exposed to Dex treatment for only 48 h. These preliminary results suggest that BRG1 plays a critical role in regulating the expression of some of the proteins whose levels are upregulated in response to Dex treatment of TM cells.

## Discussion

The primary objective of this study was to gain new molecular insights into glucocorticoid-induced cellular changes in the trabecular meshwork (TM) with a particular emphasis on actin cytoskeletal reorganization, crosslinking, and contraction, cell adhesion, extracellular matrix turnover, and actin-dependent transcriptional and chromatin remodeling activities in the context of elevated intraocular pressure (IOP) and the pathobiology of glaucoma.

**FIGURE 9:**
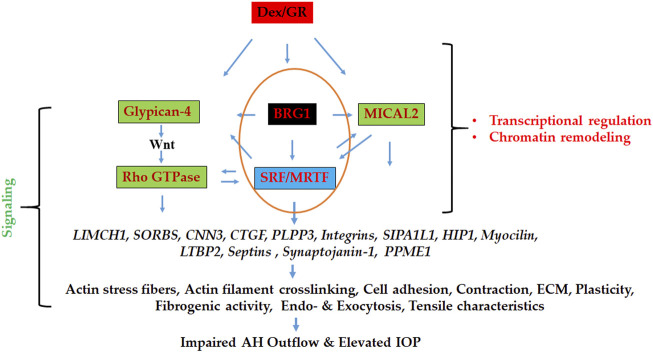
Schematic illustration of the mechanisms involved in regulating the expression of actin cytoskeletal network, contractile, cell adhesive, and vesicle trafficking proteins in Dex treated TM cells. BRG1 (regulator of chromatin remodeling), MICAL2 (regulator of nuclear actin depolymerization), and Glypican-4 (regulator of Wnt/PCP signaling) dependent pathways appear to collectively influence gene expression *via* stimulating SRF/MRTF-A transcriptional activity (a known master regulator of actin cytoskeletal and contractile protein expression) under these conditions, thereby playing a potential role in modulation of aqueous humor outflow and intraocular pressure. GR: Glucocorticoid receptor.

Our approach of using proteomics analysis to investigate dexamethasone (Dex)-induced changes in the nuclear protein profile of human TM cells enabled the identification of proteins that are not only involved in regulating actomyosin dynamics but also in the activation of chromatin remodeling and transcriptional activities. Specifically, this study identified several proteins involved in the regulation of actomyosin contraction, actin cytoskeletal crosslinking and bundling, cell adhesion, and vesicle trafficking, and perhaps even more importantly, revealed that enhancement of chromatin remodeling and transcriptional activity very likely plays a role in upregulating the expression of actin cytoskeletal and actin cytoskeletal regulatory proteins in Dex treated TM cells. These results infer that Dex treatment of human TM cells triggers changes in the levels of nucleocytoskeletal, contractile and cell adhesive proteins, and activities of actin-dependent, BRG1 mediated chromatin remodeling and actin regulated nuclear MICAL2 activity, and these molecular mechanisms acting upstream perhaps interdependently together with other regulatory mechanisms (e.g., Glypican-4 regulated Wnt/PCP signaling) controlling SRF/MRTF-A transcriptional activity, a known master regulator of the actin cytoskeletal and contractile protein gene expression in smooth muscle-like tissues (Kuwahara et al., 2005; Miano et al., 2007; Small et al., 2010). Figure 9 depicts the schematic illustration of the proposed Dex effects on the activation of molecular mechanisms acting downstream to the glucocorticoid receptors in inducing the actin cytoskeletal and cell adhesive protein gene expression and organization in TM cells in the context of the known effects of Dex on the aqueous humor outflow and intraocular pressure.

Ocular hypertension owing to an increase in resistance to aqueous humor (AH) outflow through the trabecular pathway, is a consistent finding in glucocorticoid-induced glaucoma (Clark and Wordinger, 2009; Roberti et al., 2020). Moreover, the etiology of glucocorticoid-induced IOP resembles the pathobiology of primary open-angle glaucoma in humans (Bermudez et al., 2017a). Therefore, there is a great deal of interest in understanding the pathobiology of steroid-induced IOP and glaucoma. It is well recognized that glucocorticoid-induced IOP is associated with changes in actin cytoskeletal organization, actin crosslinking, cell-ECM adhesion, cell–cell junctions, ECM accumulation, and cell stiffness in TM cells (Underwood et al., 1999; Raghunathan et al., 2015; Bermudez et al., 2017a; Filla et al., 2017). Signaling (including integrins, Rho and Rac GTPases, syndican-4 and Wnt) (Filla et al., 2011; Fujimoto et al., 2012; Sugali et al., 2021), transcriptional (e.g., Yap/Taz) (Peng et al., 2018), and epigenetic (e.g., DNA methylation) (Matsuda et al., 2015) pathways are reported to be involved in some of these glucocorticoid-induced cellular and IOP changes. Our understanding of the identity of molecular mechanisms underlying the regulation of actin cytoskeletal reorganization and assembly in TM cells by glucocorticoids however, remains elusive (Bermudez et al., 2017a). Glucocorticoids are thought to mediate their biological effects predominantly through influencing gene expression (Rhen and Cidlowski, 2005). While Dex has been shown to modulate gene expression of proteins involved in the regulation of actin cytoskeletal organization, including the actins, myosins, integrins, and actin interacting proteins (Bermudez et al., 2017a), the effector pathways and mechanisms downstream of the glucocorticoid receptor that mediates these changes in TM cells remain to be identified.

Using proteomics analysis to determine the effects of Dex on the nuclear protein profile of TM cells, an approach not previously utilized, we identified consistent changes in the levels of key proteins in multiple strains of human TM cells. Our studies identified several differentially expressed proteins involved in actin cytoskeletal changes, contraction, actin bundling, cell adhesion, and endocytosis and exocytosis, in Dex treated human TM cells. Interestingly, some of these proteins, including SORBS2 (ArgBP2) (Bollinger et al., 2012), CCN2 (CTGF) (Shan et al., 2017), PLPP3, and CNN3 have also been reported to be elevated in Dex treated TM total cell lysates (Shan et al., 2017). The concentration of glucocorticoids used in published literature involving TM cells ranges from 10^−4^ to 10^−7^ M, with treatment length spanning from a few days to several weeks (Tripathi et al., 1989; Bermudez et al., 2017b; Faralli et al., 2019). In our study, the use of a Dex concentration of 0.5 µM Dex for a maximum period of 7 days in culture did not have any adverse effect on cell survival or result in any detectable cell morphological changes by phase-contrast imaging (Supplementary Figure S7). To our surprise, however, an initial analysis using duplicates of a human TM cell strain and subsequent assessments of two additional TM cell strains revealed that Dex treatment for 7 days led to a consistent increase in the levels (by ≥ 2-fold) of several proteins in the nuclear fraction. This subset of upregulated targets was enriched in proteins involved in actin cytoskeletal organization, contraction, and bundling, actin stress fiber formation, cell adhesion, and vesicle trafficking. Importantly, SORBS2 (ArgBP2), LIMCH1 (LIM and Calponin Homology Domains 1), CNN3, and septins, which were found to be significantly elevated in Dex treated human TM cells in our study, have been shown to bind to and regulate actin crosslinking and contraction of actin stress fibers (Wang et al., 1997; Anekal et al., 2015; Lin et al., 2017; Spiliotis and Nakos, 2021). The levels of SORBS1 (CAP/Ponsin), SORBS3 (Vinexin), tensin-1, SI1LI (*SIPA1L1*), and zyxin (ZYX), which are involved in cell adhesion and actin cytoskeletal organization, were also elevated in TM cells in response to Dex treatment (Chen et al., 2002; Wang and Gilmore, 2003; Rao and Maddala, 2009; Liu et al., 2016; Ichikawa et al., 2017).

Interestingly, this study also revealed an increase in Dex treated TM cells of the levels of integrin αV (*ITGAV*) and β5 (*ITGB5*), which are well-characterized receptors of ECM proteins and regulators of cell adhesion and actin cytoskeletal organization (Filla et al., 2017). Activation of αvβ3 and increased levels of β3 integrin were also previously reported to regulate actin crosslinking and CLANS formation in Dex treated TM cells and regulate AH outflow (Filla et al., 2011; Filla et al., 2017). Several of these previously described proteins involved in actin cytoskeletal organization and crosslinking were significantly elevated in Dex treated TM cells in our studies during both the 5- and 7-day treatment period.

A noteworthy point is that the finding of Dex-mediated increases in expression of proteins involved in actin cytoskeletal organization and cell adhesion was consistently detected in nuclear extracts across multiple strains of TM cells. We have performed analyses to validate the purity of the nuclear fraction isolated from TM cells and confirmed that there is little to no contamination from the cytosolic compartment. Moreover, we observed that some of the actin cytoskeleton interacting proteins upregulated by Dex localized to both the cytosolic and nuclear fractions in TM cells. This is not entirely surprising given that several actin-interacting proteins, actin and cell adhesion proteins were detected in the nuclear fraction since many of these proteins are recognized to shuttle between the nuclear and cytosolic compartments and thus participate in gene regulation (Wang and Gilmore, 2003; Meves et al., 2009). It is not clear how this nucleocytoplasmic shuttling occurs for several of the proteins identified in this study, with some of these proteins lacking a well-defined nuclear localizing sequence. However, not all known nuclear cytoskeletal and cell adhesive proteins contain a well-defined nuclear localizing signal (Lu et al., 2021). It is also possible that some proteins detected in the nuclear fraction could bind to the external aspect of the nuclear envelope since the actin cytoskeleton is known to tether to the nuclear envelope as well. Therefore, in future studies, it is necessary to advance our insights into the mechanisms by which some of the Dex induced proteins shuttle into the nucleus in TM cells.

While the change in expression levels for some proteins exhibited a time-dependence with respect to the length of Dex treatment in TM cells (e.g., myocilin), a few proteins were upregulated as early as the 24 h treatment interval, with levels continuing to remain elevated even at the 7-day time point of Dex treatment. An example is glypican-4 (GPC4), whose levels were increased with Dex treatment at all three time points tested in this study. Glypican-4 is a well-characterized cell surface heparan sulfate proteoglycan involved in the trafficking of morphogens, including Wnt and hedgehog, and plays a vital role in their development (Song and Filmus, 2002). Glypican-4 has been shown to activate both the Wnt classical and PCP pathways (Ohkawara et al., 2003; McGough et al., 2020; Balaraju et al., 2021). Based on our observations regarding actin cytoskeletal protein levels in Dex treated TM cells, it is plausible that GPC4 activates the Wnt/PCP pathway in response to Dex treatment. Moreover, treatment of TM cells with Dex has been reported to activate the Wnt/PCP pathway and regulate actin cytoskeletal reorganization in TM cells (Yuan et al., 2013), and to inhibit Wnt/beta-catenin classical signaling activity as evidenced by elevated levels of Wnt antagonist, SFRP1 (Wang et al., 2008; Raghunathan et al., 2015). In addition to the elevation in GPC4 levels, we also detected that the levels of zyxin, a focal adhesion protein involved in the regulation of cell adhesion and actin cytoskeletal bundling, were consistently upregulated in response to Dex treatment of human TM cells. LTBP2 levels were also increased consistently with Dex treatment whose mutation has been associated with congenital glaucoma in humans and cats (Ali et al., 2009; Kuehn et al., 2016). In contrast to these proteins, the levels of TPA (tissue plasminogen activator) and neurofilament medium polypeptide were decreased consistently. Although not much is known about neurofilament medium polypeptide in TM cells, the downregulation of TPA by glucocorticoids has been documented extensively, and the increased expression of recombinant TPA has been demonstrated to lower IOP, indicating the importance of TPA in the homeostasis of IOP (Seftor et al., 1994; Candia et al., 2014).

The most interesting and intriguing finding of this study is the observation of the preferential increases in levels of proteins involved in the regulation of actin cytoskeletal dynamics, cell adhesion and contractility, and fibrogenic activity in Dex treated TM cells. Moreover, these changes appear to be largely due to their upregulation under Dex treatment since we found increased levels of some of the same proteins in the cytosolic fraction of TM cells that we analyzed for one of the TM cells strains under Dex treatment. Interestingly, many of the cytoskeletal proteins identified in this study have also been found to be differentially expressed (our unpublished studies; manuscript in preparation) in the cytoskeletome fraction obtained from Dex treated human TM cells. These findings suggest that there is a preferential upregulation of actin cytoskeletal and actin-interacting proteins in Dex treated human TM cells. This finding raises the question of why there is a preferential upregulation of expression of the proteins involved in actin cytoskeletal organization, contraction and cell adhesion under Dex treatment and what is the molecular basis underlying this response. In attempting to address these questions, we focused on the increase in levels of MICAL2, MICAL-like 2, BRG1, MTA1, and MeCP2 proteins in the nuclear fraction of Dex treated TM cells. MICAL2, a FAD-dependent monooxygenase which promotes the depolymerization of nuclear F-actin by mediating oxidation of specific methionine residues on actin, has been shown to regulate the transcriptional activity of SRF/MRTF-A (Lundquist et al., 2014). Interestingly, Dex has also been shown to increase MICAL2 expression in the brain (Juszczak and Stankiewicz, 2018).

SRF/MRTF-A is considered as a master regulator of the expression of actin cytoskeletal and contractile proteins (Olson and Nordheim, 2010; Velasquez et al., 2013; Miano et al., 2007). Moreover, BRG1, an actin and ATP dependent catalytic and essential subunit of the SWI/SNF multi-protein chromatin remodeling complex has been shown to be required for glucocorticoid-induced gene expression in various cell types (Fryer and Archer, 1998; Hoffman et al., 2018). The SWI/SNF complex plays a crucial role in regulating the chromatin state of genes and activation of gene expression (Khavari et al., 1993; Hodges et al., 2016). Interestingly, in smooth muscle-like tissues, BRG1 has been shown to interact with MRTF-A and thereby regulate SRF/MRTF-A transcriptional activity (Zhang et al., 2007; Zhou et al., 2009). Based on this collective understanding, we speculate that actin dependent, BRG1 regulated chromatin remodeling and activation of SRF/MRTF-A transcriptional activity, together with other regulatory mechanisms, including the GPC4 regulated Wnt/PCP pathway, play a key role in modulating gene expression of actin cytoskeletal, contractile and cell adhesive proteins in Dex treated human TM cells (Figure 9). Our findings of increased levels of SRF and MRTF-A and their increased nuclear localization in Dex treated TM cells, together with the ability of BRG1 deficiency to suppress levels of MICAL2 and SORBS1 and 2 and CNN3 (calponin-3) further support the importance of BRG1 and SRF/MRTF-A regulated gene expression in TM cells. Our previous studies have also uncovered the importance of SRF/MRTF-A transcriptional activity in Rho GTPase regulated TM cell contractile activity, actin cytoskeletal organization and fibrogenic activity (Pattabiraman and Rao, 2010; Pattabiraman et al., 2014). Since the SWI/SNF complex is known to not only activate but also repress the expression of certain genes (Hodges et al., 2016), the decreased levels of TPA found in across all-time Dex treated TM cell samples could be related to the gene repressive activity of this complex. In future studies, it is thus, necessary to investigate the role of BRG1, MICAL2, SFR/MRTF-A, and GPC4-regulated gene expression in TM cell actin cytoskeletal organization, contractile and fibrogenic activity and to define the mechanisms how these interactions might influence aqueous humor outflow and IOP. Finally, changes in the levels of MTA1 and MeCP2 under Dex treatment also suggest the involvement of additional chromatin regulating mechanisms in Dex induced effects in TM cells.

## Data Availability

The datasets presented in this study can be found in online repositories. The names of the repository/repositories and accession number(s) can be found below: The mass spectrometry proteomics data have been deposited to the MassIVE Dataset Summary (MassIVE MSV000088981); https://massive.ucsd.edu [doi: 10.25345/C5W08WJ0J] [dataset license: CC0 1.0 Universal (CC0 1.0)].

## Supplementary Material

Supplementary Figure S1 Dexamethasone induced actin cytoskeletal reorganization and cross-linked actin networks in human TM cells. Human TM cells treated for 7 days with 0.5 µM Dex, as described in the Methods section, revealed increased actin stress fibers (arrowheads) and cross-linked actin networks (CLANS; arrows) compared to control cells treated with ethanol (1 µl/ml media). Actin filament was stained with Phalloidin–Tetramethylrhodamine B isothiocyanate (Phalloidin-TRITC). Scale bar: Image magnification.

Supplementary Figure S2 Gene Ontology (GO) enrichment analysis of proteins exhibiting elevated levels in the nuclear fraction of human TM cells treated with Dex for 7 days. Panels **(A–C)** depict GO enrichment analyses for Biological process (using ShinyGO v0.75 online) of proteins elevated by ≥2-fold in both replicates and individual replicates (sample 1 and sample 2), respectively, where samples are derived from the same strain of TM cells treated for 7 days with Dex. Analysis was performed using a P-value cutoff (FDR) of 0.05, and results are shown for the top 30 pathways.

Supplementary Figure S3 Gene Ontology enrichment analysis of proteins exhibiting a decrease in level in the nuclear fraction of human TM cells treated with Dex for 7 days. Panels **(A–C)** depict GO enrichment analyses for biological process of proteins decreased by ≥2-fold in individual replicates (samples 1 and 2) and in both replicates, respectively, where samples are derived from the same strain of TM cells treated for 7 days with Dex. Analysis was performed using a P-value cutoff (FDR) of 0.05, and results are shown for the top 30 pathways.

Supplementary Figure S4 Gene Ontology enrichment analysis of proteins exhibiting a decrease in levels in the nuclear protein fractions derived from human TM cells treated with Dex for 7 days. GO enrichment analyses for biological process of proteins decreased by ≥2-fold in three or more samples of TM cells treated for 7 days with Dex. Analysis was performed using a P-value cutoff (FDR) of 0.05, and results are shown for the top 30 pathways.

Supplementary Figure S5 Gene Ontology enrichment analysis of proteins increased in the nuclear protein fraction derived from human TM cells treated with Dex for 5 days. GO enrichment analyses for biological process of proteins increased by ≥2-fold in three or more samples of TM cells treated for 5 days with Dex. Analysis was performed using a P-value cutoff (FDR) of 0.05, and results are shown for the top 30 pathways.

Supplementary Figure S6 Dexamethasone induced an increase in nuclear staining of MICAL2, MRTF-A, and SRF in human TM cells. Human TM cells treated with Dex (0.5 µM) for 7 days showed relatively intense staining (immunofluorescence) for MICAL2, MRTF-A, and SRF in the nucleus (arrows) compared to control cells. Scale bars indicate image magnification.

Supplementary Figure S7 Cell morphology and cell viability of human TM cells treated with dexamethasone for 7 days. Human TM cells treated with 0.5 µM Dex for 7 days were evaluated for changes in cell viability, cytotoxicity, and morphological changes, as described in the Methods section. There was no difference in cell viability (based on in vivo labeling with fluorescein diacetate-FDA (green fluorescence) and propidium iodide (red fluorescence) and cell morphology (based on phase-contrast images) compared to control cells treated with ethanol (one µl/ml media). Scale bar: Image magnification.

## Abbreviations

AH, aqueous humor; CLANS, cross-linked actin networks; Dex, dexamethasone; IOP, intraocular pressure; TM, trabecular meshwork.
