# Supplementary figures and images for: Glucocorticoids Preferentially Influence Expression of Nucleoskeletal Actin Network and Cell Adhesive Proteins in Human Trabecular Meshwork Cells
Source: Front Cell Dev Biol. 2022 Apr 26;10:886754. doi: 10.3389/fcell.2022.886754 (PMC9087352; doi:10.3389/fcell.2022.886754)

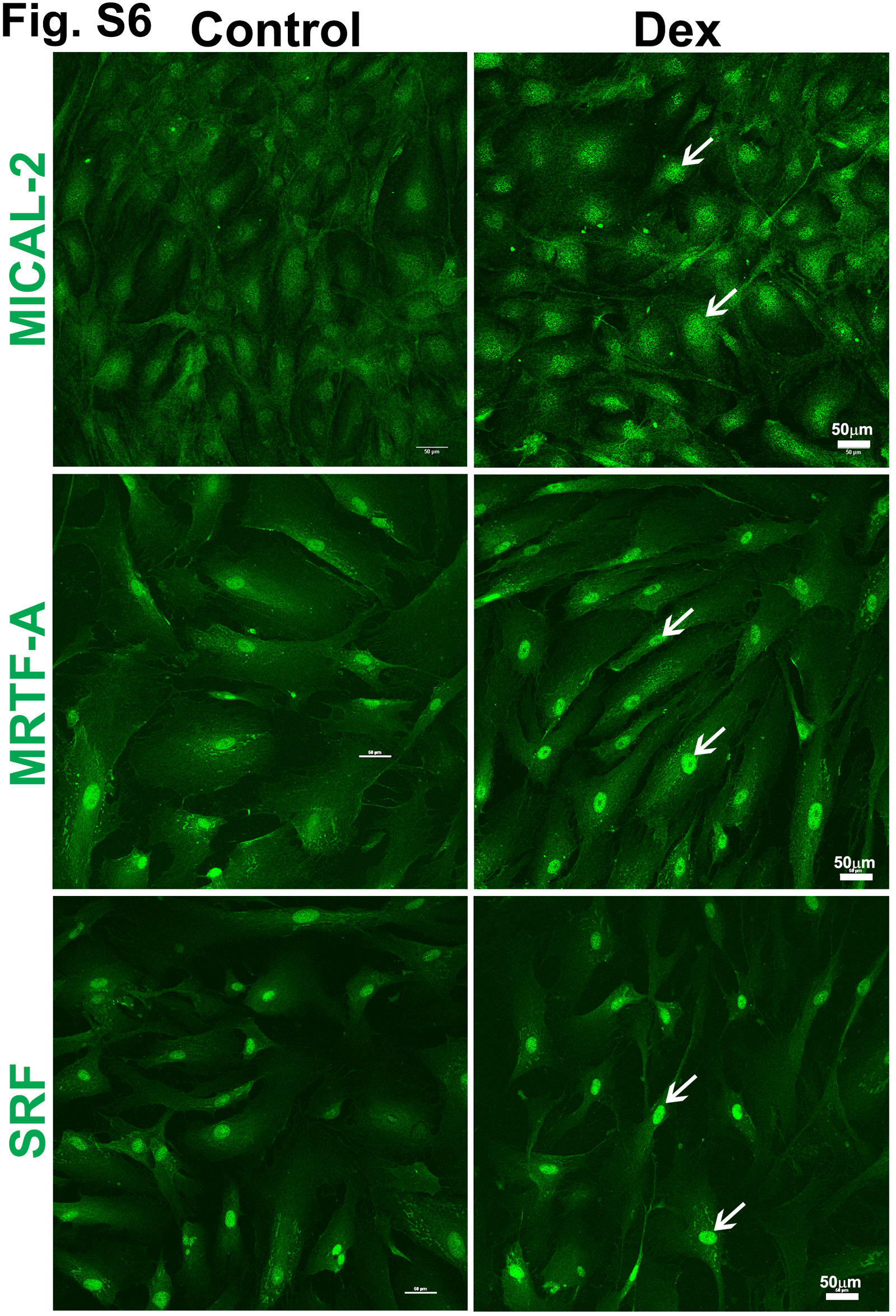

Supplement: Supplementary file 1 [file Image6.TIF]

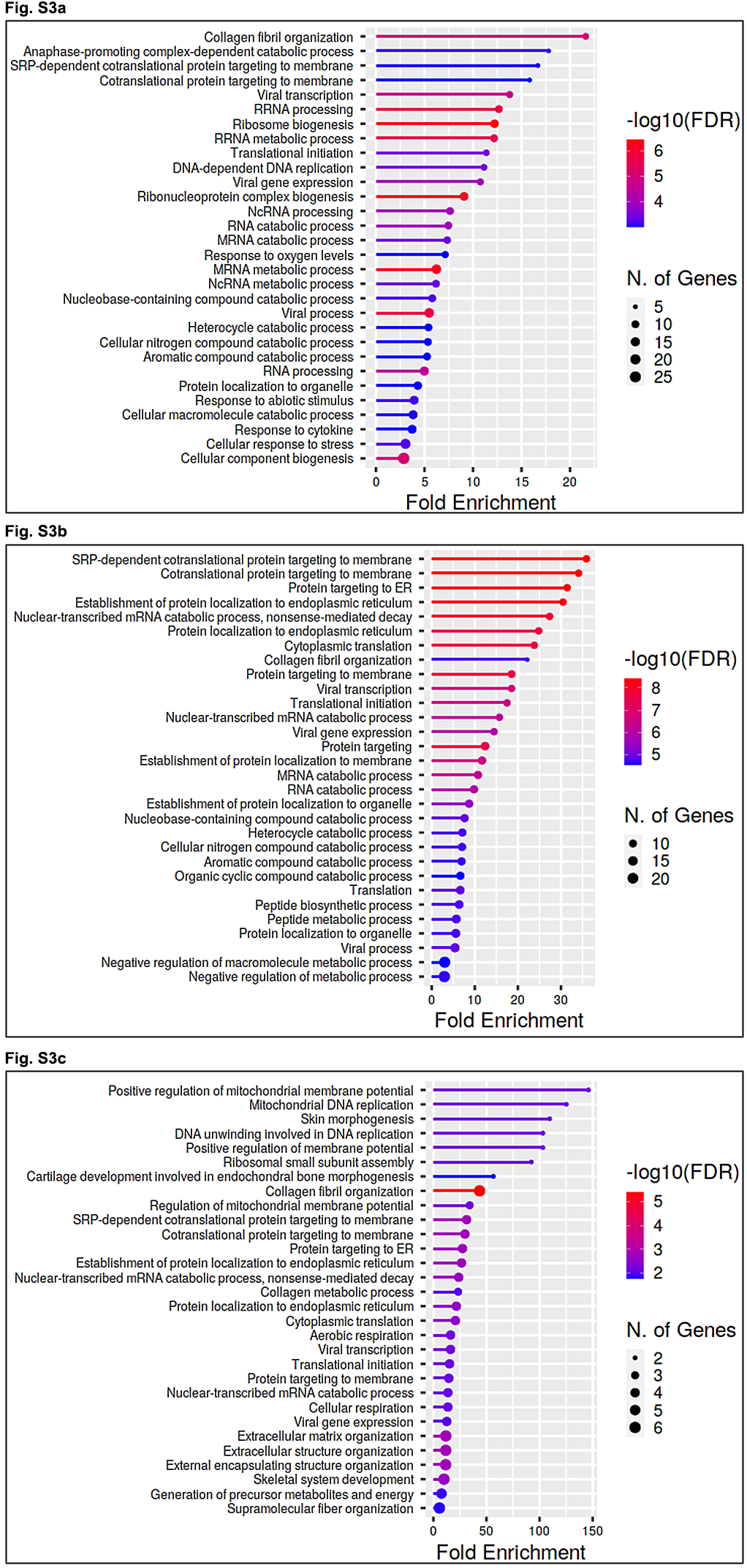

Supplement: Supplementary file 3 [file Image3.TIF]

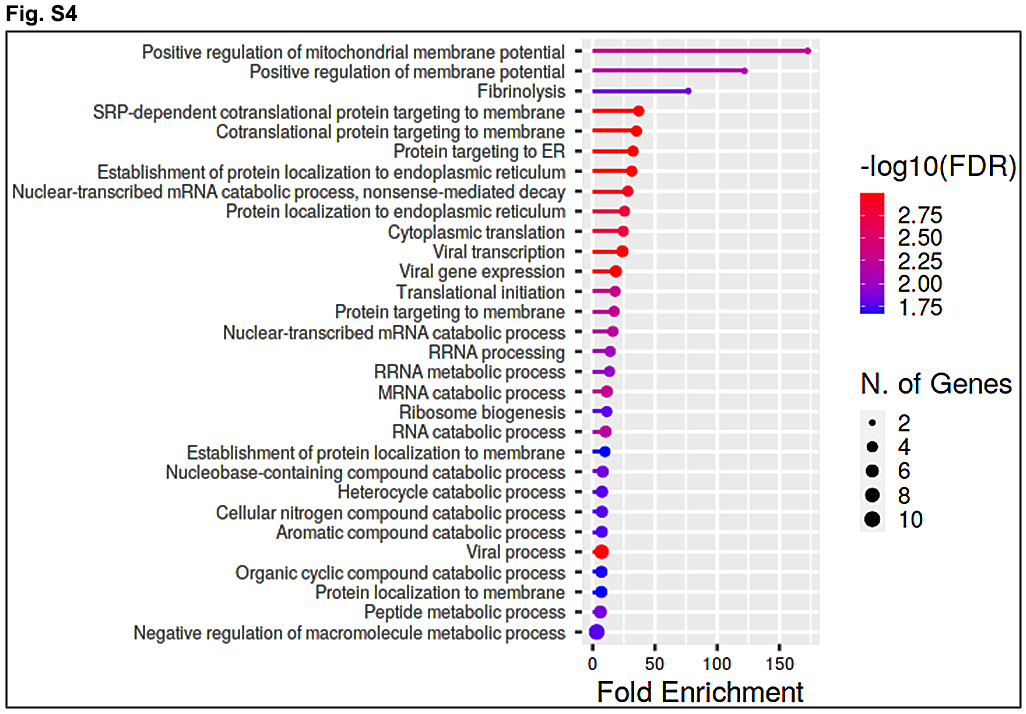

Supplement: Supplementary file 4 [file Image4.TIF]

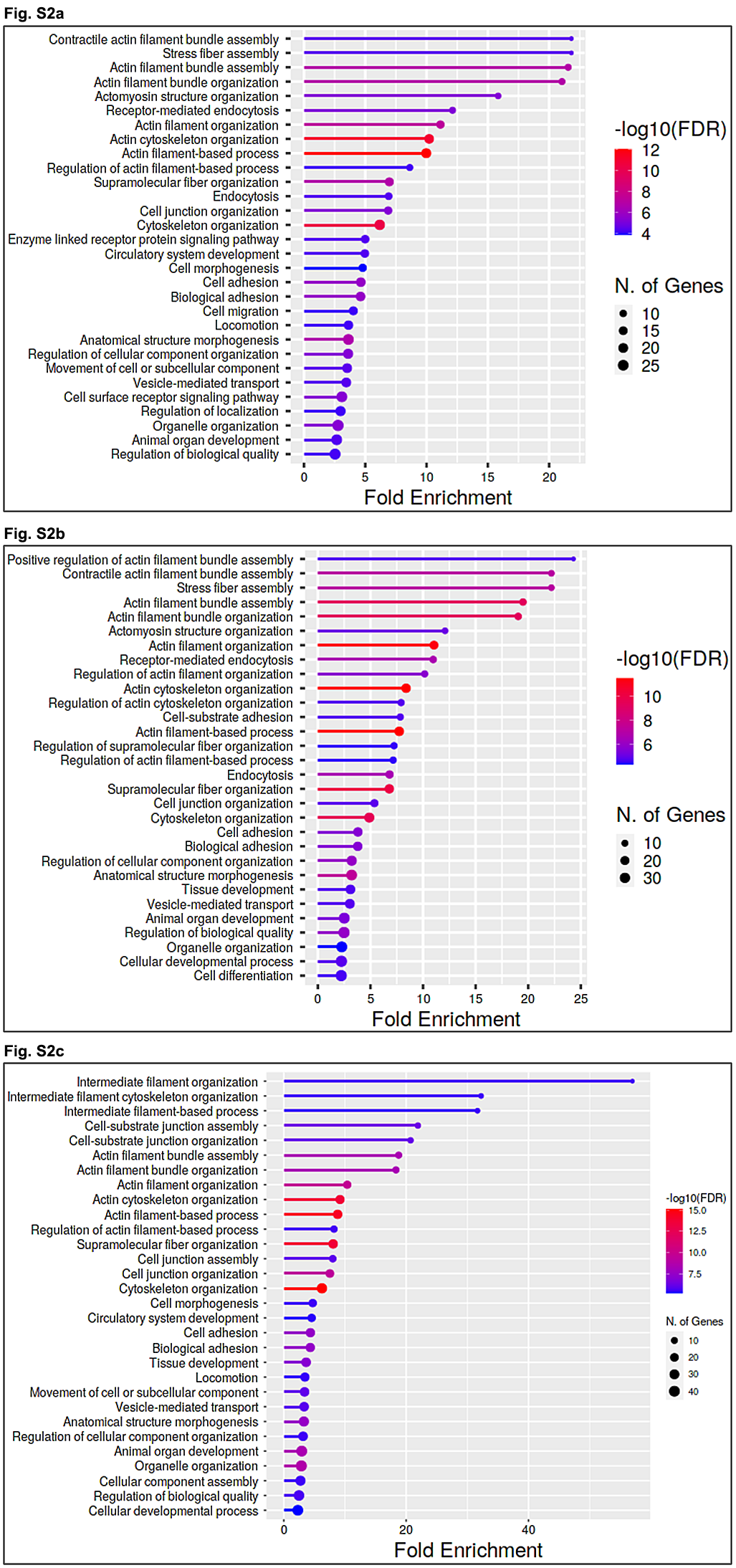

Supplement: Supplementary file 5 [file Image2.TIF]

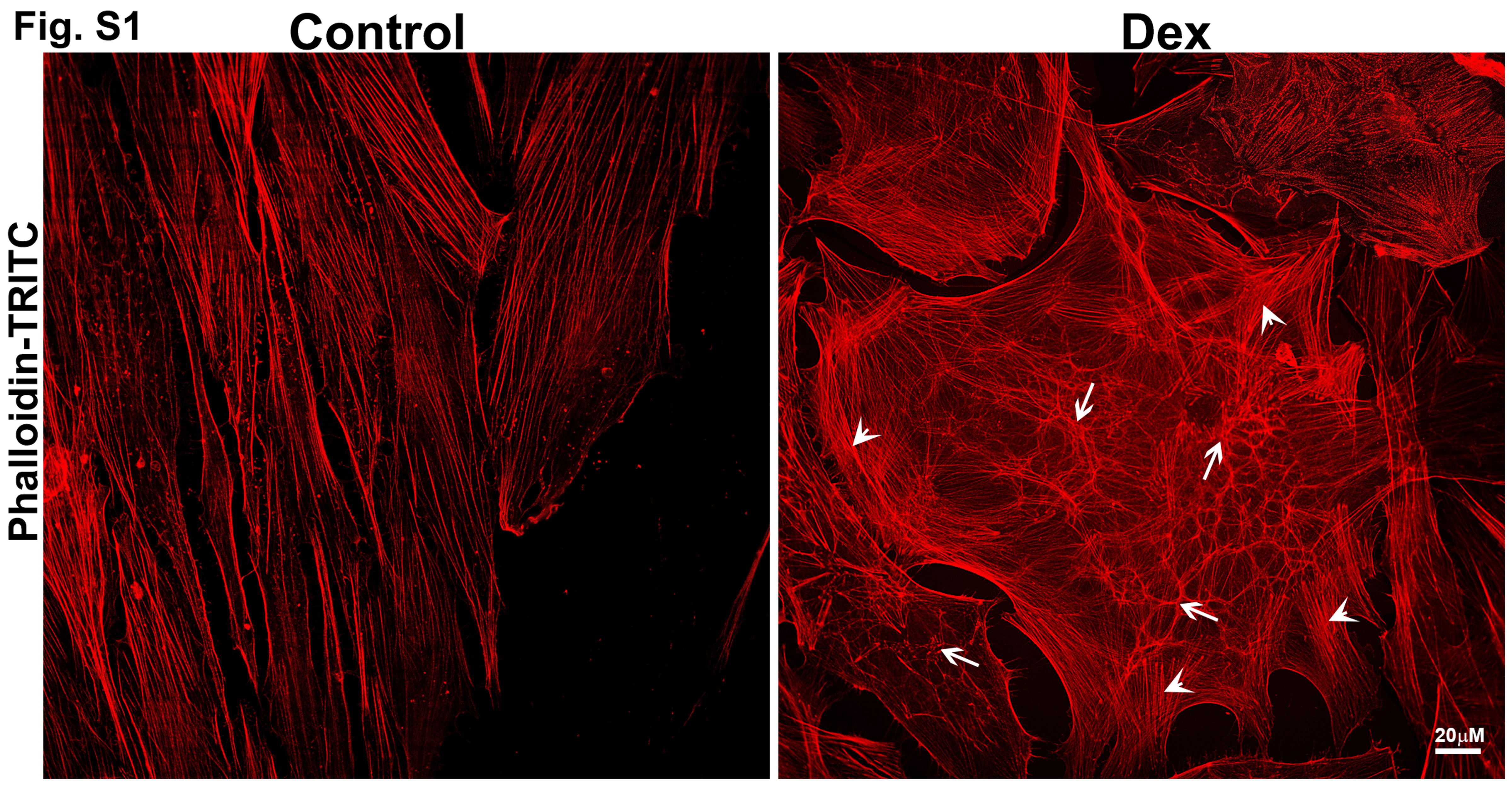

Supplement: Supplementary file 6 [file Image1.TIF]

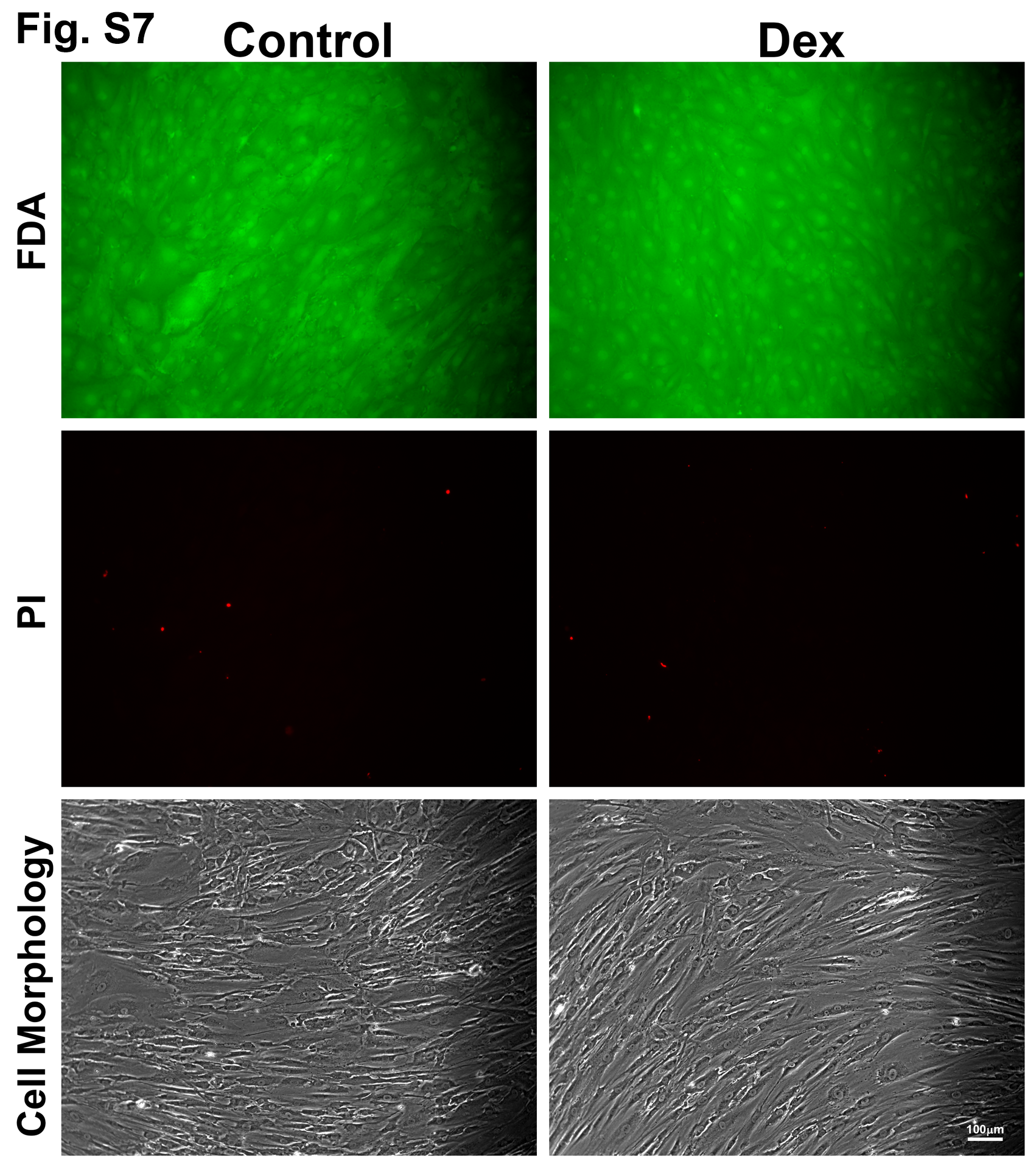

Supplement: Supplementary file 7 [file Image7.TIF]

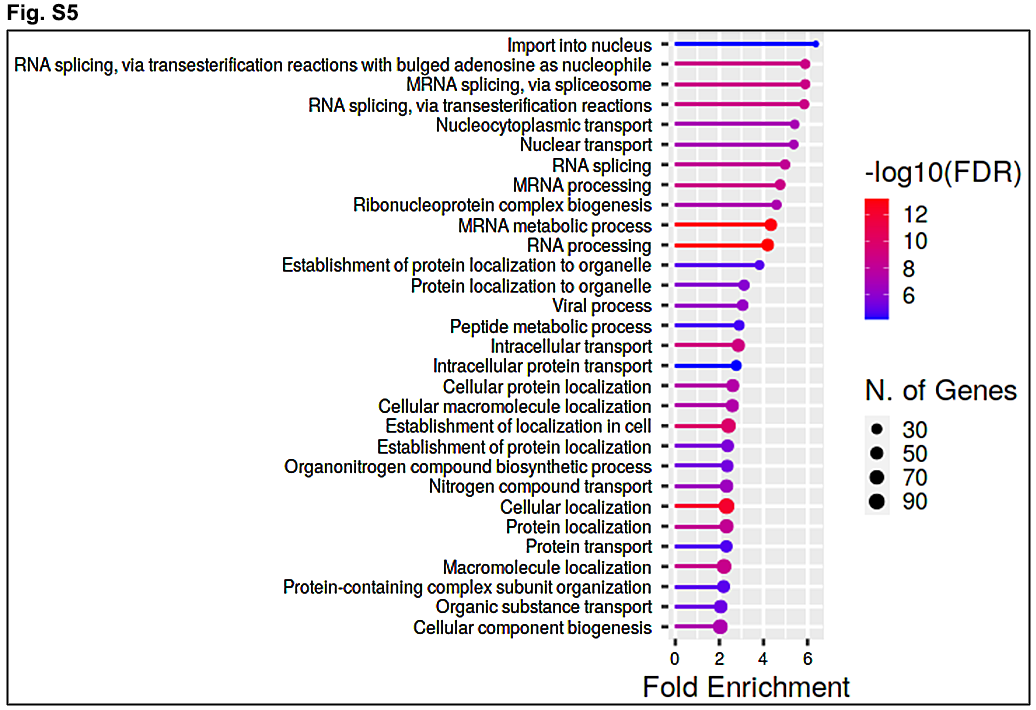

Supplement: Supplementary file 12 [file Image5.TIF]
