# Supplementary material for: Glucocorticoids Preferentially Influence Expression of Nucleoskeletal Actin Network and Cell Adhesive Proteins in Human Trabecular Meshwork Cells
Source: Front Cell Dev Biol. 2022 Apr 26;10:886754. doi: 10.3389/fcell.2022.886754 (PMC9087352; doi:10.3389/fcell.2022.886754)
Supplement: Supplementary file 2 [file Table1.DOCX]

**Table S1**. Details of antibodies used in immunoblotting (IB) and immunofluorescence (IF) analyses:

| **Antibodies** | **Cat. No.** | **Source** | **Dilution (IB)** | **Dilution (IF)** |
| --- | --- | --- | --- | --- |
| Glypican 4 Rabbit polyclonal | NBP1-87697 | Novus Biologicals, LLC; Centennial, CO, USA | 1:1000 | 1:200 |
| Glypican 4 Rabbit polyclonal | HPA030836 | Millipore Sigma, St. Louis, MO, USA | 1:1000 | 1:200 |
| SORBS1Rabbit polyclonal | NBP1-86641 | Novus Biologicals, LLC; Centennial, CO, USA | 1:1000 | 1:200 |
| Phalloidin–Tetramethyl rhodamine B isothiocyanate (TRITC) | P1951 | Millipore Sigma, St. Louis, MO, USA |  | 1:300 |
| SORBS2 Mouse monoclonal | SAB4200183 | Millipore Sigma, St. Louis, MO, USA | 1:1000 | 1:200 |
| Septin 11 Rabbit polyclonal | 440 003 | Synaptic Systems, Goettingen  Germany | 1:1000 | 1:200 |
| Synaptojanin 1 Rabbit polyclonal | NBP1-87842 | Novus Biologicals, LLC; Centennial, CO, USA |  | 1:200 |
| Acidic Calponin Rabbit polyclonal | NBP2-38643 | Novus Biologicals, LLC; Centennial, CO, USA | 1:1000 | 1:200 |
| Zyxin Antibody Rabbit polyclonal | NBP1-90349 | Novus Biologicals, LLC; Centennial, CO, USA | 1:1000 | 1:200 |
| Human LPP3 Mouse  Monoclonal | Y300150 | Applied Biological Materials Inc, Richmond, BC |  | 1:200 |
| MTA1 (D40D1) XP® Rabbit Monoclonal | 5647 | Cell Signaling Technology, Danvers, MA | 1:1000 | 1:200 |
| MeCP2 (D4F3) XP® Rabbit Monoclonal | 3456T | Cell Signaling Technology, Danvers, MA | 1:1000 | 1:200 |
| Brg1 (D1Q7F) Rabbit Monoclonal | 49360T | Cell Signaling Technology, Danvers, MA | 1:1000 | 1:200 |
| MRTF-A (G-8) Mouse Monoclonal | sc-390324 | Santa Cruz Biotechnology, Inc. Dallas, Texas | 1:1000 | 1:200 |
| MRTF-A Rabbit | Sc-398675 | Santa Cruz Biotechnology, Inc. Dallas, Texas | 1:1000 | 1:200 |
| SRF (G-20) Rabbit polyclonal | sc-335 | Santa Cruz Biotechnology, Inc. Dallas, Texas | 1:1000 |  |
| LIMCH1 Rabbit Polyclonal | HPA063840 | Millipore Sigma, St. Louis, MO, USA | 1:1000 |  |
| SEPT9 Rabbit Polyclonal | HPA042564 | Millipore Sigma, St. Louis, MO, USA | 1:1000 | 1:200 |
| Integrin alphaV Rabbit Polyclonal | AB1930 | Millipore Sigma, St. Louis, MO, USA |  | 1:200 |
| Human t-Plasminogen Activator/tPA Sheep polyclonal | AF7449 | R&D Systems, Inc. Minneapolis, MN | 1:1000 | 1:200 |
| CTGF Rabbit Polyclonal | HPA031075 | Millipore Sigma, St. Louis, MO, USA | 1:1000 | 1:200 |
| Tensin-1 Rabbit Polyclonal | NBP1-84129 | Novus Biologicals, LLC; Centennial, CO, USA | 1:1000 | 1:200 |
| Nuclear Pore Complex Proteins, Mouse  Monoclonal | ab50008 | abcam Cambridge, UK. | 1:1000 |  |
| GAPDH Mouse Monoclonal | 60004-1 | Proteintech Group, Chicago, IL | 1:1000 |  |
| Histone H2B (V119) Rabbit Polyclonal | 8135S | Cell Signaling Technology, Danvers, MA | 1:1000 |  |
| Myocilin Rabbit Polyclonal |  | Dan Stamer, Duke University | 1:1000 |  |
| Vinculin mouse monoclonal | V9131 | Sigma/Aldrich, St. Louis, MO |  | 1:200 |
| Deoxyribonuclease I, Alexa Fluor™ 594 Conjugate | D12372 | Molecular Probes, Inc.  Eugene, OR |  | 1:200 |
| Propidium Iodide | P4170 | Sigma/Aldrich, St. Louis, MO |  | 1:2000 |
| Hoechst 33258, pentahydrate  (bis-benzimide) | H21491 | Molecular Probes, Inc. / Thermo Fisher Scientific.  Eugene, OR |  | 1:1000 |

Secondary antibodies for immunofluorescence analyses:

| **Antibodies** | **Cat. No.** | **Source** | **Dilution** |
| --- | --- | --- | --- |
| Alexa Fluor^TM^ 488 goat anti-Rabbit IgG | A11077 | Invitrogen / Thermo Fisher Scientific. Rockford, IL | 1:200 |
| Alexa Fluor^TM^ 568 goat anti-mouse IgG | A11004 | Invitrogen / Thermo Fisher Scientific. Rockford, IL | 1:200 |

Secondary antibodies for immunoblot analyses:

| **Antibodies** | **Cat. No.** | **Source** | **Dilution** |
| --- | --- | --- | --- |
| Peroxidase AffiniPure Goat Anti-Rabbit IgG (H+L) | 111-035-144 | Jackson ImmunoResearch Inc, West Grove, PA | 1:5000 |
| Goat anti-Mouse IgG (H+L) Secondary Antibody, HRP | 31430 | Invitrogen / Thermo Fisher Scientific. Rockford, IL | 1:5000 |
