# Supplementary material for: Glucocorticoids Preferentially Influence Expression of Nucleoskeletal Actin Network and Cell Adhesive Proteins in Human Trabecular Meshwork Cells
Source: Front Cell Dev Biol. 2022 Apr 26;10:886754. doi: 10.3389/fcell.2022.886754 (PMC9087352; doi:10.3389/fcell.2022.886754)
Supplement: Supplementary file 8 [file Table2.DOCX]

**Table S2:** Dexamethasone induced increase (by ≥2-fold) in the levels of nuclear fraction proteins in duplicate samples of the same strain of human TM cells.

| **Accession** | **Protein Description** | **TM-1** | **TM-2** |
| --- | --- | --- | --- |
| ABCD3 | ATP-binding cassette sub-family D member 3 |  | * |
| ACADV | Very long-chain specific acyl-CoA dehydrogenase, mitochondrial | * |  |
| ACSL1 | Long-chain-fatty-acid--CoA ligase 1 | * | * |
| ACTN1 | Alpha-actinin-1 |  | * |
| ADDA | Alpha-adducin | * | * |
| ADDG | Gamma-adducin | * |  |
| AHNK | Neuroblast differentiation-associated protein AHNAK | * |  |
| AKAP2 | A-kinase anchor protein 2 | * | * |
| ALG5 | Dolichyl-phosphate beta-glucosyltransferase | * |  |
| AOFA | Amine oxidase [flavin-containing] A | * | * |
| AP2A2 | AP-2 complex subunit alpha-2 | * |  |
| APOB | Apolipoprotein B-100 | * | * |
| CADH2 | Cadherin-2 | * | * |
| CAND1 | Cullin-associated NEDD8-dissociated protein 1 |  | * |
| CAVN2 | Caveolae-associated protein 2 | * | * |
| CCN2 | CCN family member 2 (CTGF) | * | * |
| CD14 | Monocyte differentiation antigen CD14 | * | * |
| CD59 | CD59 glycoprotein | * | * |
| CD81 | CD81 antigen | * |  |
| CNN2 | Calponin-2 |  | * |
| CNN3 | Calponin-3 |  | * |
| CO8A1 | Collagen alpha-1(VIII) chain |  | * |
| CP1B1 | Cytochrome P450 1B1 | * | * |
| CYBR1 | Cytochrome b reductase 1 | * |  |
| DBNL | Drebrin-like protein | * |  |
| DD19A | ATP-dependent RNA helicase DDX19A | * |  |
| DSG1 | Desmoglein-1 |  | * |
| EPS15 | Epidermal growth factor receptor substrate 15 | * | * |
| EPS8 | Epidermal growth factor receptor kinase substrate 8 | * | * |
| FERM2 | Fermitin family homolog 2 | * |  |
| FHL1 | Four and a half LIM domains protein 1 | * |  |
| FLNA | Filamin-A |  | * |
| FLNB | Filamin-B | * | * |
| G6PI | Glucose-6-phosphate isomerase | * | * |
| GBB2 | Guanine nucleotide-binding protein G(I)/G(S)/G(T) subunit beta-2 | * |  |
| GDIB | Rab GDP dissociation inhibitor beta |  | * |
| GLU2B | Glucosidase 2 subunit beta | * |  |
| GNAI3 | Guanine nucleotide-binding protein G(i) subunit alpha | * |  |
| GPC4 | Glypican-4 | * | * |
| HBA | Hemoglobin subunit alpha |  | * |
| HTRA1 | Serine protease HTRA1 | * | * |
| ITAV | Integrin alpha-V | * | * |
| ITB5 | Integrin beta-5 | * | * |
| ITM2B | Integral membrane protein 2B | * |  |
| ITSN1 | Intersectin-1 | * | * |
| KANK2 | KN motif and ankyrin repeat domain-containing protein 2 |  | * |
| KCD12 | BTB/POZ domain-containing protein KCTD12 | * | * |
| LAMC1 | Laminin subunit gamma-1 | * |  |
| LDHA | L-lactate dehydrogenase A chain | * | * |
| LDHB | L-lactate dehydrogenase B chain | * | * |
| LIMC1 | LIM and calponin homology domains-containing protein 1 | * | * |
| MFGM | Lactadherin | * | * |
| MICA2 | [F-actin]-monooxygenase MICAL2 | * |  |
| MYO6 | Unconventional myosin-VI | * |  |
| MYOC | Myocilin | * | * |
| NEP | Neprilysin | * |  |
| NEXN | Nexilin | * | * |
| PACN3 | Protein kinase C and casein kinase substrate in neurons protein 3 | * |  |
| PAWR | PRKC apoptosis WT1 regulator protein | * | * |
| PDLI5 | PDZ and LIM domain protein 5 |  | * |
| PDLI7 | PDZ and LIM domain protein 7 | * | * |
| PGK1 | Phosphoglycerate kinase 1 | * |  |
| PGS2 | Decorin | * | * |
| PIP | Prolactin-inducible protein |  | * |
| PLEC | Plectin |  | * |
| PLPP3 | Phospholipid phosphatase 3 | * | * |
| PPME1 | Protein phosphatase methylesterase 1 | * | * |
| PYGB | Glycogen phosphorylase, brain form | * | * |
| S39AE | Zinc transporter ZIP14 | * |  |
| SEP11 | Septin-11 | * | * |
| SETLP | Protein SETSIP | * |  |
| SI1L1 | Signal-induced proliferation-associated 1-like protein 1 | * | * |
| SPTB2 | Spectrin beta chain, non-erythrocytic 1 |  | * |
| SRBS1 | Sorbin and SH3 domain-containing protein 1 (CAP/Ponsin) | * | * |
| SRBS2 | Sorbin and SH3 domain-containing protein 2 (ArgBP2) | * | * |
| STOM | Erythrocyte band 7 integral membrane protein | * | * |
| SYLC | Leucine--tRNA ligase, cytoplasmic | * |  |
| SYNJ1 | Synaptojanin-1 | * | * |
| SYNPO | Synaptopodin | * | * |
| TENS1 | Tensin-1 | * | * |
| TIMP3 | Metalloproteinase inhibitor 3 | * | * |
| TPIS | Triosephosphate isomerase | * |  |
| UGPA | UTP--glucose-1-phosphate uridylyltransferase |  | * |
| VATH | V-type proton ATPase subunit H |  | * |
| VIME | Vimentin | * | * |
| ZYX | Zyxin | * |  |
| ABLM1 | Actin-binding LIM protein 1 |  | * |
| CNTN1 | Contactin-1 | * |  |
| DESP | Desmoplakin |  | * |
| DPYL2 | Dihydropyrimidinase-related protein 2 |  | * |
| FBN1 | Fibrillin-1 |  | * |
| FHL2 | Four and a half LIM domains protein 2 |  | * |
| FILA2 | Filaggrin-2 | * |  |
| NRP2 | Neuropilin-2 | * |  |
| PARVA | Alpha-parvin |  | * |
| SNX9 | Sorting nexin-9 | * |  |
| AMPN | Aminopeptidase N |  |  |
| DEST | Destrin |  |  |
| DHC24 | Delta(24)-sterol reductase |  |  |
| HIP1 | Huntingtin-interacting protein 1 |  |  |
| PEPL1 | Probable aminopeptidase NPEPL1 |  |  |
| SART3 | Squamous cell carcinoma antigen recognized by T-cells 3 |  |  |
| SYQ | Glutamine--tRNA ligase |  |  |
| TES | Testin |  |  |
| VINEX | Vinexin |  |  |

**Footnote:** “*” indicates significant (P<0.05) increase in level of identified protein in nuclear extracts from Dex treated (7days) human TM cells relative to control human TM cells. Lanes highlighted with gray identify proteins whose levels were elevated in both samples.
