# Supplementary material for: Glucocorticoids Preferentially Influence Expression of Nucleoskeletal Actin Network and Cell Adhesive Proteins in Human Trabecular Meshwork Cells
Source: Front Cell Dev Biol. 2022 Apr 26;10:886754. doi: 10.3389/fcell.2022.886754 (PMC9087352; doi:10.3389/fcell.2022.886754)
Supplement: Supplementary file 9 [file Table5.DOCX]

**Table S5:** Dexamethasone induced increase in the levels of nuclear protein fraction proteins by two or more folds in three or more samples of human TM cells treated with Dex for 5 days.

| **Accession** | **Description** |
| --- | --- |
| 1433F | 14-3-3 protein eta |
| 1433G | 14-3-3 protein gamma |
| 4F2 | 4F2 cell-surface antigen heavy chain |
| ACINU | Apoptotic chromatin condensation inducer in the nucleus |
| ADA10 | Disintegrin and metalloproteinase domain-containing protein 10 |
| ADNP | Activity-dependent neuroprotector homeobox protein |
| ADRM1 | Proteasomal ubiquitin receptor ADRM1 |
| ADT2 | ADP/ATP translocase 2 |
| AFAD | Afadin |
| AFAM | Afamin |
| AFF4 | AF4/FMR2 family member 4 |
| AGAP3 | Arf-GAP with GTPase, ANK repeat and PH domain-containing protein 3 |
| AGFG1 | Arf-GAP domain and FG repeat-containing protein 1 |
| AGO2 | Protein argonaute-2 |
| AIMP2 | Aminoacyl tRNA synthase complex-interacting multifunctional protein 2 |
| AK17A | A-kinase anchor protein 17A |
| AKP13 | A-kinase anchor protein 13 |
| AMPD2 | AMP deaminase 2 |
| APOB | Apolipoprotein B-100 |
| AQR | RNA helicase aquarius |
| AT2C1 | Calcium-transporting ATPase type 2C member 1 |
| AT5F1 | ATP synthase F(0) complex subunit B1, mitochondrial |
| ATPA | ATP synthase subunit alpha, mitochondrial |
| ATPB | ATP synthase subunit beta, mitochondrial |
| BBX | HMG box transcription factor BBX |
| BCL6 | B-cell lymphoma 6 protein |
| BD1L1 | Biorientation of chromosomes in cell division protein 1-like 1 |
| CA2D2 | Voltage-dependent calcium channel subunit alpha-2/delta-2 |
| CAV1 | Caveolin-1 |
| CCAR1 | Cell division cycle and apoptosis regulator protein 1 |
| CCD17 | Coiled-coil domain-containing protein 17 |
| CCD9B | Coiled-coil domain-containing protein 9B |
| CCN1 | CCN family member 1 (CYR61) |
| CCN2 | CCN family member 2 (CTGF) |
| CD166 | CD166 antigen |
| CD59 | CD59 glycoprotein |
| CDC27 | Cell division cycle protein 27 homolog |
| CHD1 | Chromodomain-helicase-DNA-binding protein 1 |
| CIZ1 | Cip1-interacting zinc finger protein |
| CNN1 | Calponin-1 |
| CNN3 | Calponin-3 |
| CNTN1 | Contactin-1 |
| COF1 | Cofilin-1 |
| CP1B1 | Cytochrome P450 1B1 |
| CPSF3 | Cleavage and polyadenylation specificity factor subunit 3 |
| CRTAP | Cartilage-associated protein |
| CUL1 | Cullin-1 |
| CYTSB | Cytospin-B |
| DAF | Complement decay-accelerating factor |
| DAZP1 | DAZ-associated protein 1 |
| DDX56 | Probable ATP-dependent RNA helicase DDX56 |
| DHCR7 | 7-dehydrocholesterol reductase |
| DHE3 | Glutamate dehydrogenase 1, mitochondrial |
| DIDO1 | Death-inducer obliterator 1 |
| DIP2B | Disco-interacting protein 2 homolog B |
| DKC1 | H/ACA ribonucleoprotein complex subunit DKC1 |
| DMD | Dystrophin |
| DSE | Dermatan-sulfate epimerase |
| E41L1 | Band 4.1-like protein 1 |
| EBP2 | Probable rRNA-processing protein EBP2 |
| EI2BE | Translation initiation factor eIF-2B subunit epsilon |
| EIF3J | Eukaryotic translation initiation factor 3 subunit J |
| ELYS | Protein ELYS |
| EMAL1 | Echinoderm microtubule-associated protein-like 1 |
| EMC8 | ER membrane protein complex subunit 8 |
| EP2A2 | Laforin, isoform 9 |
| EPS15 | Epidermal growth factor receptor substrate 15 |
| ERGI1 | Endoplasmic reticulum-Golgi intermediate compartment protein 1 |
| ERPG3 | Chimeric ERCC6-PGBD3 protein |
| EXOS4 | Exosome complex component RRP41 |
| FACE1 | CAAX prenyl protease 1 homolog |
| FBN2 | Fibrillin-2 |
| FILA2 | Filaggrin-2 |
| FKBP5 | Peptidyl-prolyl cis-trans isomerase FKBP5 |
| FLOT1 | Flotillin-1 |
| FZD6 | Frizzled-6 |
| GBB1 | Guanine nucleotide-binding protein G(I)/G(S)/G(T) subunit beta-1 |
| GBB2 | Guanine nucleotide-binding protein G(I)/G(S)/G(T) subunit beta-2 |
| GBG12 | Guanine nucleotide-binding protein G(I)/G(S)/G(O) subunit gamma-12 |
| GLSK | Glutaminase kidney isoform, mitochondrial |
| GNL3 | Guanine nucleotide-binding protein-like 3 |
| GPC4 | Glypican-4 |
| GPNMB | Transmembrane glycoprotein NMB |
| GSTP1 | Glutathione S-transferase P |
| H2A1A | Histone H2A type 1-A |
| HIP1 | Huntingtin-interacting protein 1 |
| HM13 | Minor histocompatibility antigen H13 |
| IBP7 | Insulin-like growth factor-binding protein 7 |
| IDHC | Isocitrate dehydrogenase [NADP] cytoplasmic |
| IF1AX | Eukaryotic translation initiation factor 1A, X-chromosomal |
| IF5A1 | Eukaryotic translation initiation factor 5A-1 |
| IMA3 | Importin subunit alpha-3 |
| INT13 | Integrator complex subunit 13 |
| INT7 | Integrator complex subunit 7 |
| ITM2B | Integral membrane protein 2B |
| ITPI2 | Protein ITPRID2 |
| ITSN1 | Intersectin-1 |
| K1C9 | Keratin, type I cytoskeletal 9 |
| K2C1 | Keratin, type II cytoskeletal 1 |
| KAD2 | Adenylate kinase 2, mitochondrial |
| KCAB2 | Voltage-gated potassium channel subunit beta-2 |
| KIF2A | Kinesin-like protein KIF2A |
| KRI1 | Protein KRI1 homolog |
| LAR4B | La-related protein 4B |
| LETM1 | Mitochondrial proton/calcium exchanger protein |
| LOXL1 | Lysyl oxidase homolog 1 |
| LPAR1 | Lysophosphatidic acid receptor 1 |
| LTBP2 | Latent-transforming growth factor beta-binding protein 2 |
| M2OM | Mitochondrial 2-oxoglutarate/malate carrier protein |
| MAGT1 | Magnesium transporter protein 1 |
| MAOX | NADP-dependent malic enzyme |
| MAVS | Mitochondrial antiviral-signaling protein |
| MDGA1 | MAM domain-containing glycosylphosphatidylinositol anchor protein 1 |
| MDHM | Malate dehydrogenase, mitochondrial |
| MECP2 | Methyl-CpG-binding protein 2 |
| MILK2 | MICAL-like protein 2 |
| MIO | GATOR complex protein MIOS |
| MMTA2 | Multiple myeloma tumor-associated protein 2 |
| MOXD1 | DBH-like monooxygenase protein 1 |
| MPCP | Phosphate carrier protein, mitochondrial |
| MRC2 | C-type mannose receptor 2 |
| MTA1 | Metastasis-associated protein MTA1 |
| MYH2 | Myosin-2 |
| NB5R3 | NADH-cytochrome b5 reductase 3 |
| NBN | Nibrin |
| NEMF | Nuclear export mediator factor NEMF |
| NFIA | Nuclear factor 1 A-type |
| NIPA | Nuclear-interacting partner of ALK |
| NNTM | NAD(P) transhydrogenase, mitochondrial |
| NOLC1 | Nucleolar and coiled-body phosphoprotein 1 |
| NPC1 | NPC intracellular cholesterol transporter 1 |
| NSRP1 | Nuclear speckle splicing regulatory protein 1 |
| NUCKS | Nuclear ubiquitous casein and cyclin-dependent kinase substrate 1 |
| NUMA1 | Nuclear mitotic apparatus protein 1 |
| NUP50 | Nuclear pore complex protein Nup50 |
| OBSL1 | Obscurin-like protein 1 |
| ODPB | Pyruvate dehydrogenase E1 component subunit beta, mitochondrial |
| OGT1 | UDP-N-acetylglucosamine--peptide N-acetylglucosaminyltransferase 110 kDa subunit |
| OTUD4 | OTU domain-containing protein 4 |
| P4R3A | Serine/threonine-protein phosphatase 4 regulatory subunit 3A |
| PARL | Presenilins-associated rhomboid-like protein, mitochondrial |
| PARP9 | Protein mono-ADP-ribosyltransferase PARP9 |
| PDLI1 | PDZ and LIM domain protein 1 |
| PERI | Peripherin |
| PGK1 | Phosphoglycerate kinase 1 |
| PHB | Prohibitin |
| PHB2 | Prohibitin-2 |
| PHF5A | PHD finger-like domain-containing protein 5A |
| PININ | Pinin |
| PLPP3 | Phospholipid phosphatase 3 |
| PLRG1 | Pleiotropic regulator 1 |
| PNO1 | RNA-binding protein PNO1 |
| PP1RA | Serine/threonine-protein phosphatase 1 regulatory subunit 10 |
| PRP19 | Pre-mRNA-processing factor 19 |
| PRP8 | Pre-mRNA-processing-splicing factor 8 |
| PRS7 | 26S proteasome regulatory subunit 7 |
| PSA3 | Proteasome subunit alpha type-3 |
| PSB7 | Proteasome subunit beta type-7 |
| PSDE | 26S proteasome non-ATPase regulatory subunit 14 |
| PSMD2 | 26S proteasome non-ATPase regulatory subunit 2 |
| RAB2A | Ras-related protein Rab-2A |
| RAB6A | Ras-related protein Rab-6A |
| RAI3 | Retinoic acid-induced protein 3 |
| RB11A | Ras-related protein Rab-11A |
| RBM3 | RNA-binding protein 3 |
| RED1 | Double-stranded RNA-specific editase 1 |
| RFIP3 | Rab11 family-interacting protein 3 |
| RFOX1 | RNA binding protein fox-1 homolog 1 |
| RFTN2 | Raftlin-2 |
| RFX1 | MHC class II regulatory factor RFX1 |
| RL12 | 60S ribosomal protein L12 |
| RL18A | 60S ribosomal protein L18a |
| RL23 | 60S ribosomal protein L23 |
| RL28 | 60S ribosomal protein L28 |
| RL34 | 60S ribosomal protein L34 |
| RL37A | 60S ribosomal protein L37a |
| RM01 | 39S ribosomal protein L1, mitochondrial |
| RM21 | 39S ribosomal protein L21, mitochondrial |
| RM24 | 39S ribosomal protein L24, mitochondrial |
| RNPS1 | RNA-binding protein with serine-rich domain 1 |
| RPGF6 | Rap guanine nucleotide exchange factor 6 |
| RS30 | 40S ribosomal protein S30 |
| RUXF | Small nuclear ribonucleoprotein F |
| S12A4 | Solute carrier family 12 member 4 |
| S35B2 | Adenosine 3'-phospho 5'-phosphosulfate transporter 1 |
| SAFB2 | Scaffold attachment factor B2 |
| SBDS | Ribosome maturation protein SBDS |
| SCNNA | Amiloride-sensitive sodium channel subunit alpha |
| SEPR | Prolyl endopeptidase FAP |
| SEPT2 | Septin-2 |
| SF01 | Splicing factor 1 |
| SF3A1 | Splicing factor 3A subunit 1 |
| SF3B1 | Splicing factor 3B subunit 1 |
| SF3B2 | Splicing factor 3B subunit 2 |
| SFXN1 | Sideroflexin-1 |
| SLAI2 | SLAIN motif-containing protein 2 |
| SLU7 | Pre-mRNA-splicing factor SLU7 |
| SMAD2 | Mothers against decapentaplegic homolog 2 |
| SMC1A | Structural maintenance of chromosomes protein 1A |
| SMC6 | Structural maintenance of chromosomes protein 6 |
| SMCA4 | Transcription activator BRG1 |
| SMD2 | Small nuclear ribonucleoprotein Sm D2 |
| SMD3 | Small nuclear ribonucleoprotein Sm D3 |
| SMHD1 | Structural maintenance of chromosomes flexible hinge domain-containing protein 1 |
| SO6A1 | Solute carrier organic anion transporter family member 6A1 |
| SPCS2 | Signal peptidase complex subunit 2 |
| SPT2 | Protein SPT2 homolog |
| SQOR | Sulfide:quinone oxidoreductase, mitochondrial |
| SRBS2 | Sorbin and SH3 domain-containing protein 2 (ArgBP2) |
| STK25 | Serine/threonine-protein kinase 25 |
| STML2 | Stomatin-like protein 2, mitochondrial |
| STOM | Erythrocyte band 7 integral membrane protein |
| SYNJ1 | Synaptojanin-1 |
| SYTC | Threonine--tRNA ligase 1, cytoplasmic |
| TAOK1 | Serine/threonine-protein kinase TAO1 |
| TB10A | TBC1 domain family member 10A |
| THOC2 | THO complex subunit 2 |
| TM165 | Transmembrane protein 165 |
| TM9S1 | Transmembrane 9 superfamily member 1 |
| TMED2 | Transmembrane emp24 domain-containing protein 2 |
| TMED9 | Transmembrane emp24 domain-containing protein 9 |
| TOR4A | Torsin-4A |
| TOX4 | TOX high mobility group box family member 4 |
| TPR | Nucleoprotein TPR |
| TPRN | Taperin |
| TRM1L | TRMT1-like protein |
| U5S1 | 116 kDa U5 small nuclear ribonucleoprotein component |
| VAS1 | V-type proton ATPase subunit S1 |
| VINEX | Vinexin |
| VWA8 | von Willebrand factor A domain-containing protein 8 |
| VWF | von Willebrand factor |
| WDR59 | GATOR complex protein WDR59 |
| YES | Tyrosine-protein kinase Yes |
| ZBTB4 | Zinc finger and BTB domain-containing protein 4 |
| ZC11A | Zinc finger CCCH domain-containing protein 11A |
| ZCHC8 | Zinc finger CCHC domain-containing protein 8 |
| ZFHX3 | Zinc finger homeobox protein 3 |
| ZYX | Zyxin |

**Footnote**: The levels of all the listed proteins were increased by ≥2-fold in 5day Dex treated samples compared to their respective controls.
