# Supplementary material for: Glucocorticoids Preferentially Influence Expression of Nucleoskeletal Actin Network and Cell Adhesive Proteins in Human Trabecular Meshwork Cells
Source: Front Cell Dev Biol. 2022 Apr 26;10:886754. doi: 10.3389/fcell.2022.886754 (PMC9087352; doi:10.3389/fcell.2022.886754)
Supplement: Supplementary file 10 [file Table3.DOCX]

**Table S3:** Dexamethasone induced decrease (by ≥2-fold) in the levels of identified proteins in nuclear fractions prepared from duplicate cultures of the same strain of human TM cells.

| **Accession** | **Description** |
| --- | --- |
| CO1A1 | Collagen alpha-1(I) chain |
| CO1A2 | Collagen alpha-2(I) chain |
| CO3A1 | Collagen alpha-1(III) chain |
| ECI2 | Enoyl-CoA delta isomerase 2, mitochondrial |
| FKB11 | Peptidyl-prolyl cis-trans isomerase FKBP11 |
| GT251 | Procollagen galactosyltransferase 1 |
| HMGA1 | High mobility group protein HMG-I/HMG-Y |
| ISG15 | Ubiquitin-like protein ISG15 |
| LONM | Lon protease homolog, mitochondrial |
| LOXL2 | Lysyl oxidase homolog 2 |
| NDUB3 | NADH dehydrogenase [ubiquinone] 1 beta subcomplex subunit 3 |
| NNTM | NAD(P) transhydrogenase, mitochondrial |
| P5CR1 | Pyrroline-5-carboxylate reductase 1, mitochondrial |
| PLCB4 | 1-phosphatidylinositol 4,5-bisphosphate phosphodiesterase beta-4 |
| PSA7 | Proteasome subunit alpha type-7 |
| QCR2 | Cytochrome b-c1 complex subunit 2, mitochondrial |
| RL12 | 60S ribosomal protein L12 |
| RSSA | 40S ribosomal protein SA |
| SERPH | Serpin H1 |
| SSBP | Single-stranded DNA-binding protein, mitochondrial |
| STML2 | Stomatin-like protein 2, mitochondrial |
| TINAL | Tubulointerstitial nephritis antigen-like |
| TP53B | TP53-binding protein 1 |

**Footnote:** All the listed proteins were significantly (P<0.05) decreased in Dex treated (7day) samples compared to control samples
