# Supplementary material for: Glucocorticoids Preferentially Influence Expression of Nucleoskeletal Actin Network and Cell Adhesive Proteins in Human Trabecular Meshwork Cells
Source: Front Cell Dev Biol. 2022 Apr 26;10:886754. doi: 10.3389/fcell.2022.886754 (PMC9087352; doi:10.3389/fcell.2022.886754)
Supplement: Supplementary file 11 [file Table4.DOCX]

**Table S4:** Dexamethasone induced decrease (by ≥2-fold) in the levels of identified proteins in nuclear fractions derived from three or more samples of human TM cells.

| **Accession** | **Description** |
| --- | --- |
| CO1A1 | Collagen alpha-1(I) chain |
| ECI2 | Enoyl-CoA delta isomerase 2, mitochondrial |
| H2A1A | Histone H2A type 1-A |
| ITA3 | Integrin alpha-3 |
| LOXL2 | Lysyl oxidase homolog 2 |
| M2OM | Mitochondrial 2-oxoglutarate/malate carrier protein |
| MPRI | Cation-independent mannose-6-phosphate receptor |
| NNTM | NAD(P) transhydrogenase, mitochondrial |
| PSA7 | Proteasome subunit alpha type-7 |
| QCR2 | Cytochrome b-c1 complex subunit 2, mitochondrial |
| RL12 | 60S ribosomal protein L12 |
| RL34 | 60S ribosomal protein L34 |
| RS28 | 40S ribosomal protein S28 |
| SFXN1 | Sideroflexin-1 |
| SP100 | Nuclear autoantigen Sp-100 |
| STML2 | Stomatin-like protein 2, mitochondrial |
| TPA | Tissue-type plasminogen activator |
| UT14A | U3 small nucleolar RNA-associated protein 14 homolog A |

**Footnote:** All the listed proteins were significantly (P<0.05) decreased in 7day Dex treated samples relative to control samples.
